# NBS1-CtIP–mediated DNA end resection suppresses cGAS binding to micronuclei

**DOI:** 10.1093/nar/gkac079

**Published:** 2022-02-22

**Authors:** Salim Abdisalaam, Shibani Mukherjee, Souparno Bhattacharya, Sharda Kumari, Debapriya Sinha, Janice Ortega, Guo-Min Li, Hesham A Sadek, Sunil Krishnan, Aroumougame Asaithamby

**Affiliations:** Department of Radiation Oncology, University of Texas Southwestern Medical Center, Dallas, TX 75390, USA; Department of Radiation Oncology, University of Texas Southwestern Medical Center, Dallas, TX 75390, USA; Department of Radiation Oncology, University of Texas Southwestern Medical Center, Dallas, TX 75390, USA; Department of Radiation Oncology, University of Texas Southwestern Medical Center, Dallas, TX 75390, USA; Department of Radiation Oncology, University of Texas Southwestern Medical Center, Dallas, TX 75390, USA; Department of Radiation Oncology, University of Texas Southwestern Medical Center, Dallas, TX 75390, USA; Department of Radiation Oncology, University of Texas Southwestern Medical Center, Dallas, TX 75390, USA; Department of Internal Medicine, University of Texas Southwestern Medical Center, Dallas, TX 75390, USA; Department of Radiation Oncology, Mayo Clinic Florida, Jacksonville, FL 32082, USA; Department of Radiation Oncology, University of Texas Southwestern Medical Center, Dallas, TX 75390, USA

## Abstract

Cyclic guanosine monophosphate–adenosine monophosphate synthase (cGAS) is activated in cells with defective DNA damage repair and signaling (DDR) factors, but a direct role for DDR factors in regulating cGAS activation in response to micronuclear DNA is still poorly understood. Here, we provide novel evidence that Nijmegen breakage syndrome 1 (NBS1) protein, a well-studied DNA double-strand break (DSB) sensor—in coordination with Ataxia Telangiectasia Mutated (ATM), a protein kinase, and Carboxy-terminal binding protein 1 interacting protein (CtIP), a DNA end resection factor—functions as an upstream regulator that prevents cGAS from binding micronuclear DNA. When NBS1 binds to micronuclear DNA via its fork-head–associated domain, it recruits CtIP and ATM via its N- and C-terminal domains, respectively. Subsequently, ATM stabilizes NBS1’s interaction with micronuclear DNA, and CtIP converts DSB ends into single-strand DNA ends; these two key events prevent cGAS from binding micronuclear DNA. Additionally, by using a cGAS tripartite system, we show that cells lacking NBS1 not only recruit cGAS to a major fraction of micronuclear DNA but also activate cGAS in response to these micronuclear DNA. Collectively, our results underscore how NBS1 and its binding partners prevent cGAS from binding micronuclear DNA, in addition to their classical functions in DDR signaling.

## Graphical Abstract

**Graphical Abstract:**
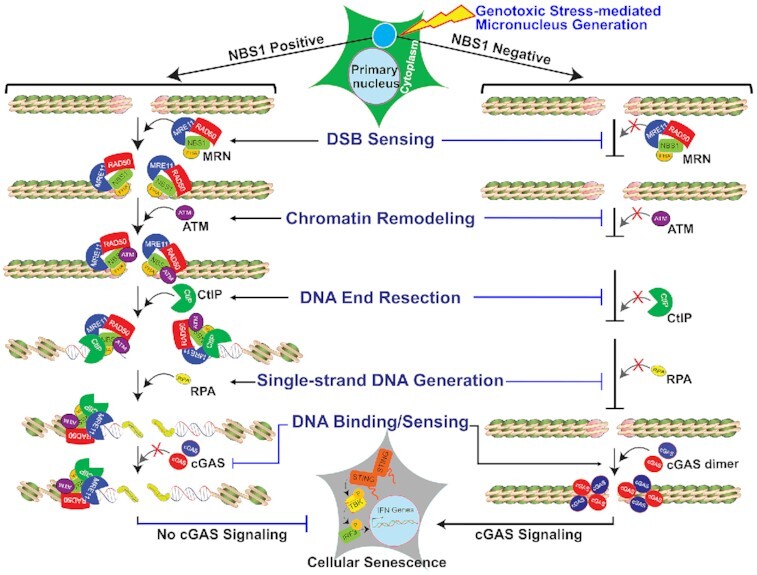
Following micronuclei generation in response to genotoxic stress, NBS1 is recruited to micronuclear DNA and that promotes CtIP-mediated end resection, resulting in the inhibition of cGAS binding to the micronuclear DNA.

## INTRODUCTION

Cyclic guanosine monophosphate–adenosine monophosphate synthase (cGAS), one of the most essential components of the innate immune system, is a cytosolic DNA sensor that is activated by binding to double-stranded DNA (dsDNA), including microbial and self-DNA. Upon binding to dsDNA, cGAS catalyzes the formation of 2′-3′-cGAMP, an atypical cyclic di-nucleotide second messenger that binds and activates stimulator of interferon genes (STING), which results in the recruitment of TANK Binding Kinase 1, the activation of the transcription factor Interferon Regulatory Factor 3 (IRF3) and the trans-activation of innate immune response genes, including type I interferon cytokines (IFN-I) (1–3). cGAS’s association with nuclear envelope–ruptured micronuclei, i.e., cytosolic chromatin fragments, results in its activation (4–9). However, multiple mechanisms, including tight tethering to chromatin, Golgi vesiculation, and phosphorylation by CDK1-Cyclin B and Aurora B kinases, have been implicated in silencing cGAS activation in mitotic cells, a phase of the cell cycle in which chromatin lacks a nuclear envelope (10–14). Additionally, cGAS’s interactions with nucleosomes and barrier-to-autointegration factor 1 suppress cGAS activation by genomic DNA in the nuclear compartment (15,16). In addition to its catalytic activities, nuclear localized cGAS suppresses DNA repair and decelerates replication forks, which leads to genomic instability, tumorigenesis and suppression of replication-associated DNA damage (17–19). However, the regulation of cGAS recruitment to micronuclear DNA remains poorly understood.

Recently, an array of mechanisms has been proposed to explain the activation of cGAS-mediated innate immune signaling due to defective DNA damage response signaling (DDR) factors (20). For example, RAD51’s and RPA’s ability to bind to nuclear single-strand DNA regulates cGAS activation by outcompeting it for cytosolic DNA (21). We have demonstrated that, by blocking nuclease activity at stalled forks and DNA double-strand breaks (DSBs), RAD51 prevents degraded fragments of DNA from triggering the STING-mediated innate immune response (22). Another study found that inhibiting ATR causes the release of damaged DNA into the cytoplasm in response to ionizing radiation, and that activates the cGAS-STING pathway (23). A recent study showed that *MLH1* deficiency leads to uncontrolled DNA excision by Exonuclease 1, which increases single-strand DNA formation, RPA exhaustion, DNA breaks, and aberrant DNA repair intermediates, resulting in the activation of the *cGAS*-STING pathway (24). Furthermore, inhibiting ATM/Chk2 leads to replication stress and the accumulation of cytosolic DNA, which subsequently activates STING signaling in certain tumors (25). These findings demonstrate that the accumulation of genomic DNA in the cytoplasm due to defective DDR functioning activates cGAS-STING signaling. In addition, recent evidence shows that Bloom syndrome protein (BLM) deficiency increases cGAS-positive micronuclei (26) and that TREX1-mediated resection of micronuclear DNA limits cGAS activation in micronuclei (27). Still, whether cGAS recruitment to micronuclear DNA is directly regulated by any DDR factor remains unknown.

Nibrin or Nijmegen breakage syndrome protein 1 (NBS1) is a multifunctional protein that forms part of the MRN (MRE11–RAD50–NBS1) complex and is one of the key DDR factors. NBS1 is the protein defective in Nijmegen breakage syndrome (NBS), a rare autosomal recessive disorder associated with immune deficiency, microcephaly, chromosomal instability, and a high frequency of multiple malignancies (28). NBS1 participates in the early sensing of DNA damage and functions as a platform for recruiting and assembling the DDR components required for the repair process (29). NBS1 directly interacts with Meiotic Recombination 11 (MRE11) (30), Ataxia Telangiectasia Mutated (ATM) (31,32), C-terminal binding protein 1 (CtBP1) interacting protein (CtIP) (33–35), and the phosphorylated H2AX (γH2AX). These NBS1 interacting proteins play key roles during different stages of DDR signaling. For example, NBS1-dependent recruitment of ATM to DSB sites not only phosphorylates NBS1 but also activates CtIP (34). CtIP binds to the N-terminal FHA‐BRCT1/2 domains of NBS1; this binding is important for DNA end resection (34,36). Apart from its nuclear functions, NBS1 is recruited to micronuclei harboring DNA damage (37), but the biological significance of NBS1’s recruitment to micronuclei is not clear.

In this study, we report that cGAS does not bind to all micronuclei with ruptured nuclear envelope and that, by promoting CtIP-mediated end resection, NBS1 inhibits cGAS binding to micronuclear DNA. We found that NBS1 not only associates with micronuclear DNA harboring DSBs, but also recruits ATM and CtIP. Subsequently, CtIP converts DSB ends into single-strand DNA (ssDNA) ends. These ssDNA ends, and possibly the modification of chromatin organization that occurs during DNA end resection, block cGAS from binding to micronuclear DNA. By using a non-denaturing BrdU immunostaining approach that detects end-resected DNA, we corroborated cGAS’s inability to interact with end-resected micronuclear DNA. Finally, we provide evidence that cells lacking NBS1 and CtIP not only recruit cGAS to a major fraction of micronuclear DNA but also activate cGAS in response to these micronuclear DNA, which results in elevated levels of immune signaling and cellular senescence. Thus, by promoting CtIP-mediated end resection, NBS1 functions as an upstream regulatory factor that prevents cGAS from binding micronuclear DNA.

## MATERIALS AND METHODS

### Cell lines

HT1080, HeLa and BEAS2B cells were obtained from the American Type Culture Collection (ATCC; USA). ATLD and HEK293 cells have been described previously (38). NBS (NBS1-deficient) and NBS cells complemented with different NBS1 constructs were a kind gift from Dr Kobayashi (Kyoto University, Japan), and have been described previously (29,30,38). HT1080 + DD-Cas9/SgTelomere RNA and HT1080-dominant negative (DN)-TRF2 (45 to 453 aa regions of TRF2) cell lines have been described previously (39). HeLa cells stably expressing shATM and shGFP RNAs have been described previously (40). MDC1 knockout and wild type mouse embryonic fibroblasts have been described previously (41,42). All cell lines were grown in standard tissue culture conditions at 5% CO_2_ and maintained in Dulbecco's modified eagle medium supplemented with 10% fetal bovine serum, 2 mM glutamine and 0.1 mM nonessential amino acids. Mycoplasma contamination was frequently tested by PCR; only mycoplasma-free cells were used for all experiments.

To make stable BEAS2B-shcGAS, BEAS2B-shNBS1, BEAS2B-shCtIP, BEAS2B-shRNF20 and BEAS2B-shCtIP + RNF20 RNA cell lines, we infected BEAS2B cells with pooled lentiviral particles carrying either cGAS, NBS1, CtIP, RNF20 or CtIP + RNF20-specific shRNAs (Sigma), then placed cells under puromycin selection (0.5 μg/ml). To make a stable tetracycline-inducible BEAS2B-shNBS1 RNA cell line, we infected BEAS2B cells with pooled lentiviral particles carrying tetracycline-inducible NBS1-specific shRNAs, then placed cells under G418 selection (500 μg/ml). To stably knock down both cGAS and NBS1 in the same cells, we infected BEAS2B cells with pooled lentiviral particles harboring both shcGAS RNA (Puromycin) and tetracycline-inducible shNBS1 RNA (G418) and selected with puromycin and G418 together. Stable clones were isolated, and the expression of cGAS, NBS1, CtIP and RNF20 was verified by western blotting.

### DNA manipulation and construction of the expression vectors

Standard molecular biology procedures were used to make all mammalian expression plasmids. Panels of human cGAS-specific shRNAs (TRCN0000128706, TRCN0000128310, TRCN0000149984, TRCN0000146282 and TRCN0000150010), human NBS1-specific shRNAs (TRCN0000295898, TRCN0000040137, TRCN0000040133, TRCN0000012671 and TRCN0000012672), human CtIP-specific shRNAs (Sigma, TRCN0000318738, TRCN0000005403, and TRCN0000005405) and human RNF20-specific shRNAs (Sigma, TRCN0000033876, TRCN0000033877 and TRCN0000033878) were purchased from Sigma. To make tetracycline-inducible shNBS1 RNA constructs, we annealed shNBS1-F1/F2/F3 and shNBS1-R1/R2/R3 primers (Supplementary Table S1) in annealing buffer (100 mM NaCl, 10 mM Tris–HCl, pH 7.4), then ligated them into AgeI-EcoRI sites of a Tet-pLKO-neo vector (was a gift from Charles Rudin; Addgene plasmid #47541). We confirmed the sequence of all constructs prior to use.

### Lentiviral production

293T cells were co-transfected with highly purified expression plasmids and pPLP2/pLP1/pVSVG by using either Lipofectamine 2000 (Invitrogen), according to manufacturer's instructions, or the calcium phosphate method described by Kwon *et al.* (43). We collected cell culture supernatant containing viral particles 72 h after the transfection, filtered it through 0.22 μm PVDF membrane filters (Millipore), and used the flowthrough to infect the cells.

### Chemicals

We used 6-thio-dG (Sigma, #1296), ATM inhibitor (Sigma, #925701–46-8), doxycycline (Sigma, #D9891), cGAS inhibitor RU.521 (AOBIOUS, #37877), Shield1 (Cheminpharma, #S1-0005), Puromycin (AdipoGen), Hygromycin (Invitrogen), G418 (Sigma Aldrich), gemcitabine (Selleck Chemical, #S1149), hydroxyurea (Sigma Aldrich, # H8627), camptothecin (Sigma Aldrich; 7689-03-4) and aphidicolin (EMD Millipore, #178273).

### Doxycycline and Shield1 treatment

(a) To induce DSBs in the telomeric DNA of HT1080-sgTelomere-DD-Cas9 cells, we co-treated cells with 500 ng/ml doxycycline (Sigma) and 1 μM Shield1 (Cheminpharma) for 24 h. Cells were then washed three times with warm PBS and allowed to recover in regular growth medium; samples were collected after 24–72 h. (b) To induce DN-TRF2 expression, we treated HT1080-DN-TRF2 cells with 1000 ng/ml doxycycline (Sigma) for 72 h. Cells were then washed three times with warm PBS and allowed to recover in regular growth medium; samples were collected at 24–74 h post-DOX withdrawal (w/d) time points. (c) To deplete NBS1, we treated cells expressing BEAS2B-shNBS1 RNA with 500 ng/ml doxycycline (Sigma) for 72 h. Cells were then treated with 6-thio-dG (3–5 μM) in the presence of 500 ng/ml doxycycline (Sigma), washed three times with warm PBS and allowed to recover in regular growth medium containing 500 ng/ml doxycycline; samples were collected at different post-DOX withdrawal time points.

### 6-thio-dG treatment

Twenty-four hours after plating, we treated cells with 2–5 μM 6-thio-dG for 72 h, washed them three times in warm PBS, and then cultured them in drug-free medium for an additional 0–10 days. Samples were collected at different post–6-thio-dG recovery times.

### Mirin treatment

Thirty-six hours after seeding, we treated cells with 10 μM mirin for 72 h together with 6-thio-dG. Then, 6-thio-dG was removed, and cells were cultured in mirin-containing medium for 0–10 days. Samples were collected at different post–6-thio-dG recovery times.

### cGAS inhibitor (RU.521) treatment

Cells were treated with 1 μM RU.521 dissolved in DMSO for the entire duration of the experiment. Fresh drug was added to the medium every 48 h for continuous inhibition of cGAS.

### ATM inhibitor (KU55933) treatment

Cells were treated with 10–20 μM KU55933 dissolved in DMSO for the entire duration of the experiment. Fresh drug was added to the medium every 24 h for continuous inhibition of ATM kinase activity.

### Ionizing radiation

We exposed exponentially growing cells to 2–5 Gy γ-rays by using a γ-irradiator (Mark 1 irradiator, JL Shepherd & Associates) as described previously (44).

### Cell extracts and western blotting

We prepared whole cell extracts by suspending cell pellets in RIPA buffer containing protease inhibitors cocktail (Calbiochem; Catalog #539133), PMSF (1:100), dithiothreitol (1:1000) and phosphatase inhibitors (Na_3_VO_4_ at 1:500 and NAF at 1:200 dilution) on ice for 30 min, followed by centrifugation at 14 000 RPM for 30 min at 4 °C to remove insoluble material. Whole-cell extracts (30–100 μg) were resolved by 6–12% SDS-PAGE, transferred onto polyvinylidene difluoride membranes and incubated with antibodies of interest.

### Antibodies used in this study

All the primary antibodies used in this study are detailed in Supplementary Table S2, including vendors' information and the application and dilution conditions. Manufacturers' validation criteria were used for the application of all antibodies. For western blotting, HRP-conjugated goat anti-rabbit and anti-mouse secondary antibodies were purchased from BioRad and used at 1:1000 dilution in 5% BSA or milk. For indirect immunofluorescence staining, fluorescent-conjugated secondary antibodies Alexa-488, Alexa-555 and Alexa-633 were purchased from Molecular Probes (Invitrogen) and used at 1:1000 dilution.

### Indirect immunostaining

Approximately 0.5–1 × 10^5^ cells were plated in a six-well plate with cover glasses and incubated for 36 h. Cells were treated with various chemicals for different time periods, as described above. Cells were fixed with 4% paraformaldehyde (PFA) for 20 min at room temperature at different post-treatment times and subjected to indirect immunofluorescence, as described previously (39). Briefly, cells were permeabilized in Triton X-100 (0.5% in PBS) on ice for 5 min, washed three times with PBS, incubated in blocking solution (5% goat serum in PBS) at room temperature for 60 min, and then incubated with primary antibodies (diluted in 5% goat serum) at room temperature for another 3 h or at 4 °C overnight. Then, cells were washed with wash buffer (1% BSA in PBS), incubated with appropriate secondary antibodies (1:1000 in 2.5% goat serum, 1% BSA, and PBS) at room temperature for 60 min, washed five times with 1% BSA, and mounted with mounting medium containing DAPI (Vectashield).

### DNA end resection assessment in micronuclei

To evaluate DNA end resection in micronuclei, we labeled cells with 30 μM BrdU in regular cell culture medium in the dark for 24–36 h. Twenty-four hours after exposure to genotoxic agents, cells were pre-extracted with CSK buffer on ice for 10 min and then fixed with 4% PFA. These cells were then subjected to indirect immunostaining using anti-BrdU antibodies (Rat), as described above.

### Image acquisition

Images were captured by using an LSM 510 Meta laser scanning confocal microscope with a 63 × 1.4 NA Plan-Apochromatic oil immersion objective. Images were taken of z-sections (15–20 sections) at 0.35-μm intervals by using the 488-nm (EGFP and Alexa 488), 543-nm (Alexa 555), 633-nm (Alexa 633) and 405-nm (for DAPI) lasers. The tube current of the 488-nm argon laser was set at 6.1 A. The laser power was typically set to 3–5% transmission with the pinhole opened to 1–2 Airy units. To enumerate individual and co-localized proteins in the micronuclei, we assembled the z-sections by using the Imaris software and analyzed them as described previously (39). We quantified individual and co-localized proteins from images of 50–1000 cells per time point from minimum three independent experiments.

### Micronuclei imaging and quantification

Cells fixed with 4% PFA were mounted with mounting solution containing DAPI (Vectashield). Images were acquired via an Axio-Invert (Zeiss) microscope using the DAPI channel, and the exposure time ranged from 400 to 500 μs per frame. We used ImageJ (NIH) to calculate micronuclei in a blinded fashion in 100–1000 cells per experimental condition in minimum three independent experiments.

### Senescence assay

Cells freshly fixed with 2% formaldehyde and 0.2% glutaraldehyde were subjected to β-galactosidase (β-gal) staining by using either β-gal staining kit (Cell Signaling), following manufacturer's instructions, or a homemade β-gal staining solution, as described previously (39). We acquired random images by using the 10X objective of a KEYENCE Microscope (BZ-X710) in a blinded manner. We counted in a blinded fashion the number of β-gal–positive cells in 20–30 random fields consisting of 1000–5000 total cells per experimental condition in minimum three independent experiments.

### Quantitative real-time polymerase chain reaction (qRT-PCR)

qRT-PCR was performed in accordance with the Minimum Information for Publication of Quantitative Real-Time PCR Experiments (MIQE) guidelines. Briefly, total RNA was isolated from mock- and -treated cells using the Qiagen RNeasy kit (217004, Qiagen), according to manufacturer's instructions. Concentration of each RNA sample was measured using the Nanodrop 1000 Spectrophotometer (Thermo Scientific). Prior to the cDNA synthesis, a DNase treatment was performed using the RQ1 RNase-free DNase according to the manufacturer's instructions (Promega). We synthesized cDNA from 1–3 μg of total RNA by using SuperScript III Reverse Transcriptase (18-080-051; Fisher Scientific) in a total volume of 20 μl, according to manufacturer's instructions. We subjected the cDNA to qRT-PCR for several genes by using the primer sets (Supplementary Table S3), CFX96 Touch Real-Time PCR Detection System (Bio Rad) and iTaq Universal SYBR Green Supermix (Bio Rad; #1725121), according to manufacturer's instructions. Relative gene expression was determined by the ΔΔCT method. The difference in cycle times, ΔCT, was determined as the difference between the tested gene of interest and the reference β-actin gene. We then obtained ΔΔCT by finding the difference between the groups. The fold change (FC) was calculated as FC = 2^–ΔΔCT^. All primers were purchased from Invitrogen. qRT-PCR assays were carried out in triplicate for each sample, and the mean value was used to calculate mRNA expression levels, as described previously (22).

### ELISA

We collected cell culture supernatant at different post-treatment times and centrifuged at 800 g for 5 min at room temperature to remove any cell debris. We then measured cytokine levels by ELISA via the Biolegend human IL-6 (Cat# 430504) kit, as per the manufacturer's instructions and as described previously (45).

### Expression and purification of human cGAS

Human cGAS–expressing plasmid construct pET28a-His-hcGAS was a kind gift from Dr Li (46). Purified plasmid was transformed into BL21(DE3)-competent cells and selected by using Kanamycin (50 μg/ml). A single bacterial colony was grown in regular LB medium containing 50 μg/ml Kanamycin at 16°C to an optical density of 0.25 at 580 nM. Protein expression was initiated by IPTG (500 ng/ml) at 16°C overnight. cGAS purification was carried out as described previously (47). Briefly, BL21(DE3) cell pellets were resuspended and lysed by sonication in 25 mM HEPES–NaOH, pH 7.9, 5% glycerol, 20 mM Imidazole, 300 mM NaCl and 0.2 mM PMSF. Lysate was clarified by centrifugation at 100 000 × g for 1 hr. Clear lysate was loaded into a 5-ml HisTrap HP (GE Healthcare) column, and, after the column was washed with 25 mM HEPES–NaOH, pH 7.9, 5% glycerol, 25 mM imidazole, 300 mM NaCl and 0.2 mM PMSF, cGAS was eluted with a gradient of 25–500 mM imidazole. Fractions containing cGAS were combined and loaded into 1 mL Heparin (GE Healthcare). After being washed with 25 mM HEPES-NaOH, pH 7.9, 5% glycerol, 0.1 mM EDTA, 500 mM NaCl, 1 mM DTT and 0.2 mM PMSF, cGAS was eluted with a gradient of 0.5–1 M NaCl. Fractions containing cGAS were combined and loaded into Superdex 200 Increase 10/300 GL (GE Healthcare) in 25 mM HEPES–NaOH, pH 7.9, 5% glycerol, 0.1 mM EDTA, 150 mM NaCl, 1 mM DTT and 0.2 mM PMSF.

### Gel shift analysis

This was performed as described previously (48). Briefly, samples were assembled in 20 μl reactions containing 10 mM HEPES–NaOH, pH 7.5, 100 mM NaCl, 1 mM DTT, 1 mM MgCl_2_, 20 μg/ml BSA, 25–50 fmol DNA and cGAS. After incubating at 25ºC for 30 min, we stopped the reactions by adding 2 μl of 50% glycerol, 0.05% bromophenol blue, 0.05% xylene cyanol and 20 mM EDTA. Samples were loaded onto 6% native polyacrylamide gel (19:1) in 0.5× TBE. Gel electrophoresis was performed at room temperature.

### Statistics

Statistical analysis was performed by using GraphPad Prism Software (version 9.0). Two-tailed unpaired Student's *t*-tests and two-way analysis of variance (ANOVA) were used for statistical analyses, and unless otherwise noted, all results represent at least three independent biological experiments and are reported as the mean ± standard deviation. GraphPad Prism (version 9.0) was used to create the graphs.

## RESULTS

### cGAS is recruited to only a sub-set of micronuclei in response to genotoxic stress

**Figure 1.**
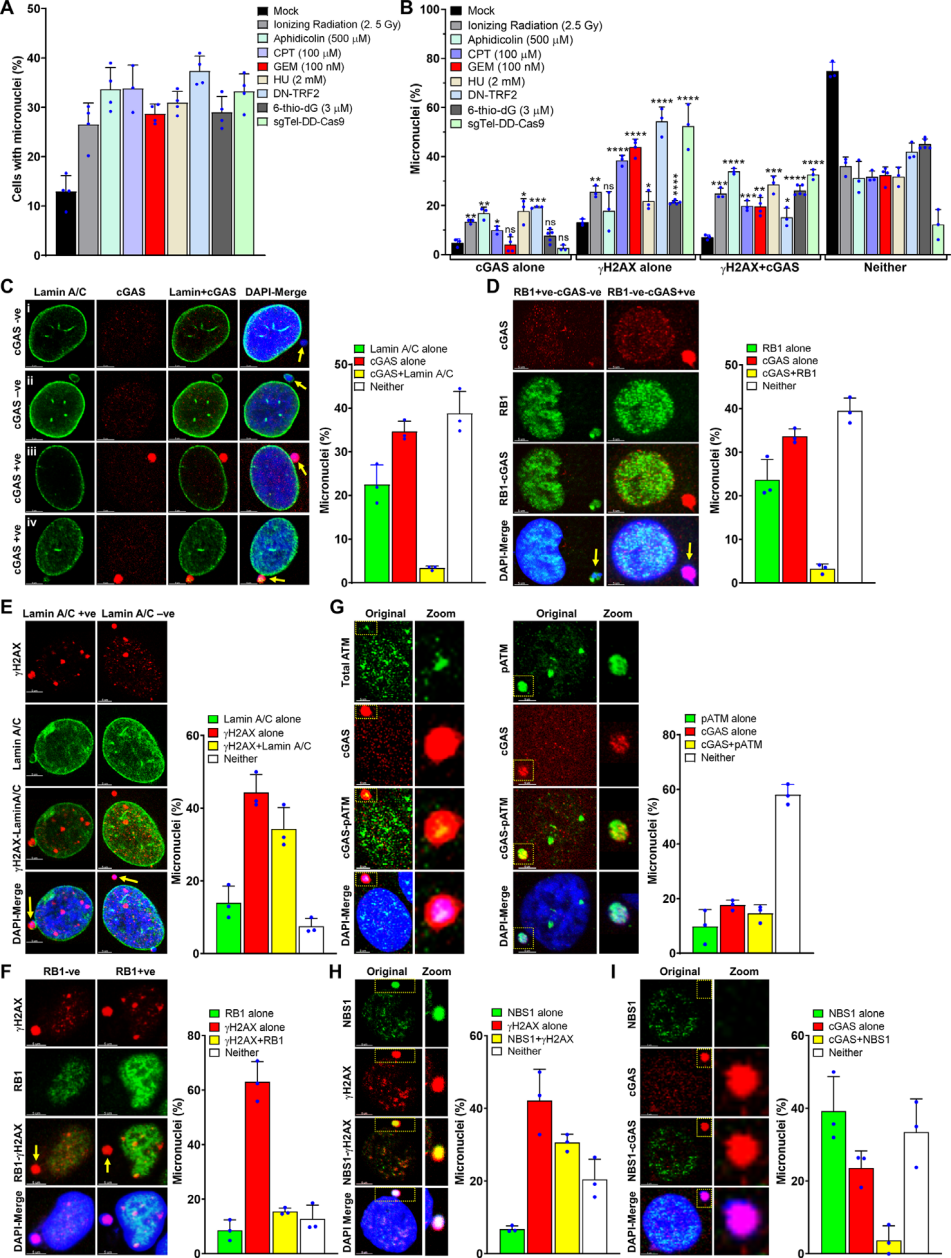
cGAS is recruited to only a sub-population of nuclear envelope–ruptured micronuclei in response to genotoxic stress. (A, B) cGAS is recruited to only a limited number of micronuclei. Bar graphs show percentages of micronuclei-positive cells (**A**) and the percentage of micronuclei harboring either cGAS alone, γH2AX alone, cGAS and γH2AX or neither (**B**) at 24–72 h after exposing human bronchial epithelial (BEAS2B) cells to either mock or different DNA damaging agents (APH/CPT/GEM/6-thio-dG); HT1080 + DN-TRF2 cells treated with doxycycline (DN-TRF2); and HT1080 + sgTel-DD-Cas9 cells concomitantly treated with doxycycline and Shield1 (sgTel-DD-Cas9). The bar graph presents the mean and STDEV from three to five independent experiments. Statistical analysis was performed by using Student's *t*-test. APH–aphidicolin; CPT–camptothecin; GEM–gemcitabine; HU–hydroxyurea; 6-thio-dG–6-thio-2′-deoxyguanine; DN-TRF2–overexpression of doxycycline-inducible dominant negative telomeric-repeat binding factor 2; sgTel-DD-Cas9–CRISPR/Cas9-mediated induction of DNA double-strand breaks in the telomeric-repeat DNA. * *P* ≤ 0.05; ** *P* ≤ 0.01; *** *P* ≤ 0.001; **** *P* ≤ 0.0001. (C, D) Not all micronuclei (MN) with ruptured nuclear envelopes (NE) recruit cGAS. Representative images show cGAS-negative and Lamin A/C–negative (**i**), cGAS-negative and Lamin A/C–positive (**ii**), cGAS-positive and Lamin A/C (NE)–negative (**iii**) and cGAS-positive and Lamin A/C (weak)–positive (**iv**) micronuclei (**C**, left panels). Bar graph shows the frequency of micronuclei harboring either Lamin A/C alone, cGAS alone, cGAS and Lamin A/C or neither in BEAS2B cells treated with 3 μM 6-thio-dG for 72 h (**C**, right). Representative images show localization of either cGAS or RB1 in micronuclei (**D**, left). Bar graph shows the frequency of micronuclei harboring either RB1 alone, cGAS alone, cGAS and RB1 or neither in BEAS2B cells treated with 3 μM 6-thio-dG (**D**, right). Bar graph presents the mean and STDEV from 150–200 cells from three independent experimental groups. (**E**) A major fraction of γH2AX-positive micronuclei are devoid of a nuclear envelope. Representative images show the presence or absence of γH2AX in Lamin A/C coating–positive and negative micronuclei (left). Bar graph shows the frequency of micronuclei harboring either Lamin A/C alone, γH2AX alone, Lamin A/C and γH2AX, or neither in BEAS2B cells treated with 3 μM 6-thio-dG for 72 h (right). Bar graph presents the mean and STDEV from three independent experimental groups. +ve–positive; -ve–negative. (**F**) Representative images show the presence or absence of γH2AX in RB1-positive and RB1-negative micronuclei (left). Bar graph shows the percentage of micronuclei harboring either γH2AX alone, RB1 alone, γH2AX and RB1 or neither in BEAS2B cells treated with 3 μM 6-thio-dG for 72 h (right). Bar graph presents the mean and STDEV from three independent experimental groups. (**G**) Recruitment and phosphorylation of ATM (pATM, S1981) in micronuclei, and a fraction of pATM co-localizes with cGAS. Representative images show co-localization of total ATM (left) and phosphorylated ATM (S1981; middle) with cGAS in micronuclei. Bar graphs show the frequency of micronuclei containing either pATM alone, cGAS alone, pATM and cGAS or neither in BEAS2B cells treated with 3 μM 6-thio-dG. Bar graph presents the mean and STDEV from three independent experimental groups. (H, I) NBS1 is recruited to γH2AX-positive micronuclei but rarely co-localizes with cGAS. Representative images show the presence of NBS1 and γH2AX (**H**, left) and only cGAS but no NBS1 (**I**, left) in micronuclei. Bar graphs show the frequency of micronuclei harboring either NBS1 alone, γH2AX alone, NBS1 and γH2AX or neither (**H**, right) and NBS1 alone, cGAS alone, NBS1 and cGAS or neither (**I**, right) in BEAS2B cells treated with 3 μM 6-thio-dG for 72 h. Bar graph presents the mean and STDEV from three independent experimental groups.

A number of studies have reported the accumulation of cGAS in nuclear envelope–ruptured micronuclei (5–9,26); however, only limited information is available on whether cGAS binds to all or to only a sub-set of these micronuclei (8). Therefore, we evaluated the efficiency of cGAS recruitment to micronuclei in human cells in response to different types of DNA damaging agents, such as ionizing radiation (IR), which is a genome-wide DSB generator; aphidicolin, camptothecin (CPT), hydroxyurea (HU) and gemcitabine (GEM), which induce replication stress; and 6-thio-2′-Dideoxyguanine (6-thio-dG), dominant negative telomeric-repeat binding factor 2 (DN-TRF2), and CRISPR/Cas9 (sgTelomere-DD-Cas9), which induce dysfunctional telomeres (39). Under similar numbers of micronuclei-generating conditions (Figure 1A), we observed that only ∼30–40% of micronuclei (cGAS alone plus cGAS and γH2AX) were positive for cGAS, regardless of how we caused genomic DNA damage (Figure 1B). We and others have recently shown that cGAS-STING signaling is activated in response to DN-TRF2, 6-thio-dG, sgTel-DD-Cas9 or ionizing radiation, among these DNA damaging agents (24,39,49). Therefore, we used DN-TRF2, 6-thio-dG, sgTel-DD-Cas9 or ionizing radiation to identify the role of DDR factors in regulating cGAS binding to micronuclear DNA for our subsequent studies.

Because cGAS is known to be recruited to nuclear envelope–ruptured micronuclei, i.e. cytosolic chromatin fragments (CCFs), we examined the relationship between cGAS recruitment and nuclear envelope coating by co-immunostaining with anti-cGAS and anti-Lamin A/C antibodies after 6-thio-dG treatment. Similar to previous reports (4–9,39), we observed that most cGAS-bound micronuclei did not have a nuclear envelope coating (Lamin A/C) (Figure 1C). Interestingly, we noticed another population (∼40%) of micronuclei that was neither Lamin A/C- nor cGAS-positive (Figure 1C). We further verified the limited recruitment of cGAS to micronuclei by co-immunostaining with RB1, another marker for intact nuclear envelope coating (Figure 1D) (6–8). As with previous reports, a major fraction of cGAS-positive micronuclei were negative for RB1, and some micronuclei were neither cGAS- nor RB1-positive (Figure 1D). Thus, only some, but not all, micronuclei without a nuclear envelope coating recruit cGAS in response to genotoxic stress.

Apart from cGAS, we also detected the phosphorylated histone 2A variant (γH2AX) signal, often used as a surrogate marker for DSBs, in a major fraction (up to 85%; γH2AX alone plus γH2AX/cGAS) of micronuclei (Figure 1B), which suggests the presence of DSBs in these micronuclei. Although most of the cGAS-positive micronuclei were positive for γH2AX foci, not all γH2AX foci–positive micronuclei recruited cGAS (Figure 1B). Furthermore, the micronuclei negative for Lamin A/C coating (Figure 1E) and RB1 (Figure 1F) were also positive for γH2AX foci. Since several DNA damage sensing, repair and signaling factors, including ATM, MDC1, NBS1 (a component of the MRN complex), BRCA1, p53 and RPA32, have been shown to localize in the micronuclei (37,50), we examined the co-localization of ATM, MDC1, MRE11, and NBS1 with cGAS in the micronuclei. Initially, we found that ∼18–35% of micronuclei were positive for phosphorylated ATM (Figure 1G), NBS1 (Figure 1H, I), MDC1 (Supplementary Figure S1A) and MRE11 (Supplementary Figure S1B) in response to 6-thio-dG. Subsequently, we noticed the co-localization of both total and phosphorylated (Serine 1981) ATM (Figure 1G) and MDC1 (Supplementary Figure S1A) with cGAS in the micronuclei. In contrast, we rarely detected co-localization of NBS1 (Figure 1I) and MRE11 (Supplementary Figure S1B) with cGAS in the same micronuclei. Thus, among the DDR factors examined, NBS1 and MRE11 do not co-localize with cGAS in the same micronuclei.

### NBS1 deficiency enhances cGAS-positive micronuclei

**Figure 2.**
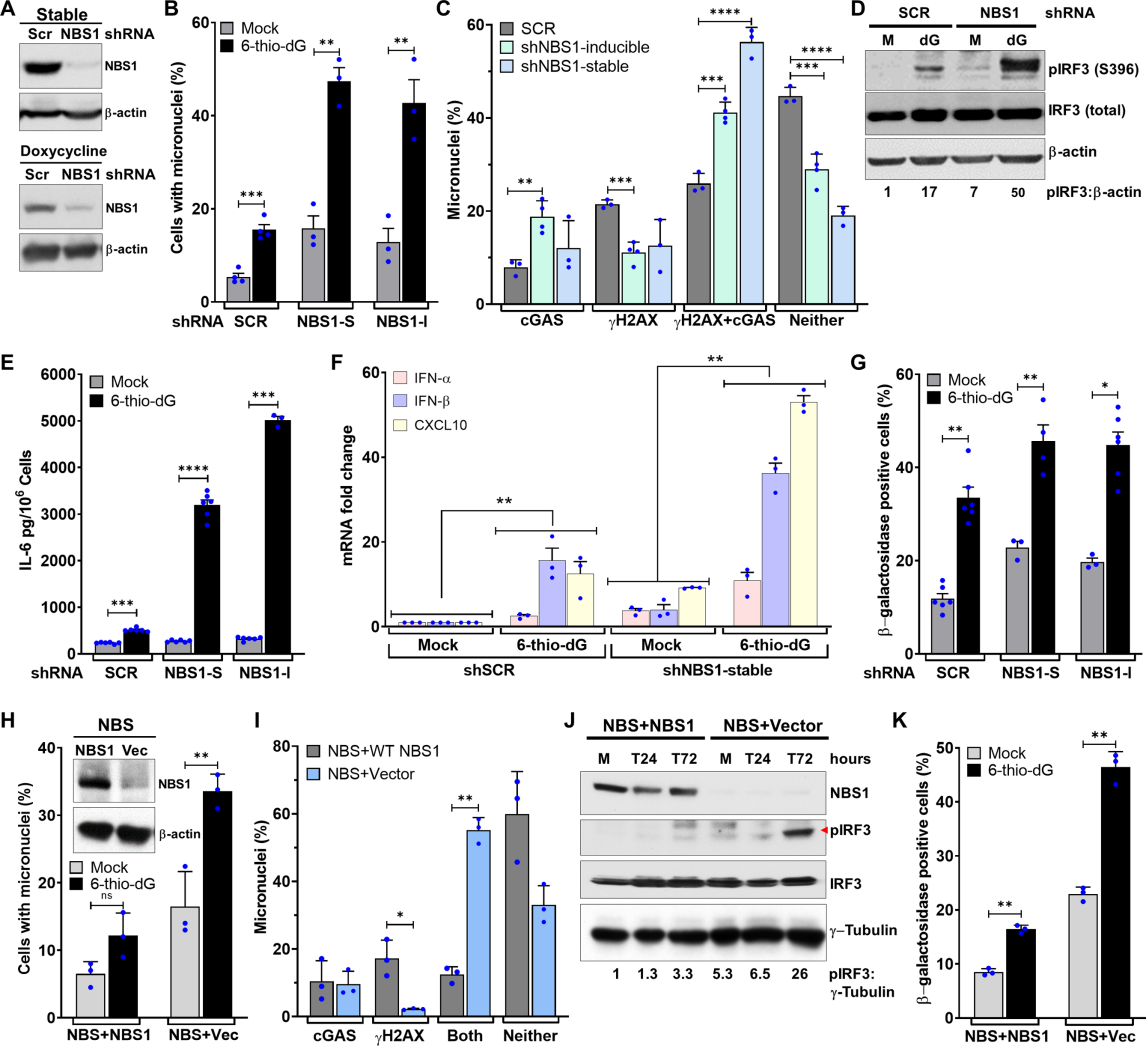
NBS1 deficiency enhances cGAS-positive micronuclei. (A–C) Stable and transient NBS1 knockdown enhances cGAS-positive micronuclei. Representative western blots show stable (top) and doxycycline (lower)-mediated NBS1 depletion in BEAS2B cells (**A**). Bar graph shows percentages of micronuclei in mock- and 3 μM 6-thio-dG (72 h)–treated BEAS2B cells stably expressing scrambled (SCR), stable (NBS1-S), and doxycycline-inducible (NBS1-I) shNBS1 RNAs (**B**). Bar graph shows the percentage of micronuclei harboring either cGAS alone, γH2AX alone, cGAS and γH2AX or neither in BEAS2B cells expressing scrambled (SCR), stable, and doxycycline-inducible shNBS1 RNAs at 72 h after 3 μM 6-thio-dG treatment (**C**). Bar graph presents the mean and STDEV from three independent experiments. Statistical analysis was performed using Student's *t*-test. (D–G) NBS1 knockdown enhances IRF3 phosphorylation, IL-6 secretion, expression of immune genes and cellular senescence. Representative western blot shows phosphorylation of IRF3 (S396; **D**), and the bar graph shows the level of IL-6 in the BEAS2B cell culture supernatant (**E**) in mock-treated cells and at 72 h after 3 μM 6-thio-dG (dG) treatment. Graph shows greater expression of immune pathway genes in shNBS1 RNA cells treated with 3 μM 6-thio-dG than in shSCR RNA cells (**F**). Bar graph shows the frequency of β-galactosidase staining–positive cells at 10 days after 3 μM 6-thio-dG treatment in shSCR, shNBS1-stable (S) and doxycycline-inducible (I) RNA-expressing BEAS2B cells (**G**). Bar graph presents the mean and STDEV from three-six independent experiments. Statistical analysis was performed using Student's *t*-test (E and G) and two-way ANOVA (F). (H, I) Elevated levels of cGAS-positive micronuclei in patient-derived NBS1-mutant (NBS) cells. Representative western blots show expression of NBS1 in NBS cells complemented with NBS1 and vector alone (vec) (**H**, inset). Bar graph shows the percentage of micronuclei in mock- and 5 μM 6-thio-dG (72 h)–treated NBS1-mutant (NBS + Vec) and NBS cells complemented with WT NBS1 (NBS + NBS1; **H**). Bar graph shows the percentage of micronuclei harboring either cGAS alone, γH2AX alone, cGAS and γH2AX or neither in NBS1-mutant (NBS + Vector) and NBS cells complemented with WT NBS1 (NBS + NBS1) at 72 h after 5 μM 6-thio-dG treatment. Bar graphs present the mean and STDEV from three independent experiments. Statistical analysis was performed using Student's *t*-test. (J, K) NBS1 deficiency enhances IRF3 phosphorylation and exacerbates premature senescence. Representative western blots show increased activation of IRF3 (S396) in NBS cells relative to NBS cells complemented with NBS1 at indicated times after 6-thio-dG treatment (**J**). NBS cells show higher levels of β-galactosidase staining than NBS + NBS1 cells 7 days after 5 μM 6-thio-dG treatment (**K**). Bar graph presents the mean and STDEV from 12–15 different fields from three independent experiments. Statistical analysis was performed using Student's *t*-test. * *P* ≤ 0.05; ** *P* ≤ 0.01; *** *P* ≤ 0.001; **** *P* ≤ 0.0001.

Because NBS1 and cGAS do not co-localize in a major fraction of micronuclei, we investigated NBS1’s involvement in cGAS recruitment to these micronuclear DNA. First, we used shNBS1 RNA (Sigma) to stably knock down NBS1 in BEAS2B cells (Figure 2A). Initially, we observed a significantly higher percentage of cells with micronuclei in shNBS1 RNA cells than in scrambled shRNA (shSCR) after 6-thio-dG treatment (*P* ≤ 0.0.002–0.0001; Figure 2B). Unexpectedly, we noticed that ∼66% of the micronuclei in shNBS1 RNA cells were positive for cGAS, but only 33% of the micronuclei in shSCR cells were positive for cGAS in response to 6-thio-dG (Figure 2C). To rule out the possibility that the increase in cGAS-positive micronuclei was associated with genomic instability due to long-term knockdown of NBS1, we used a tetracycline-inducible shNBS1 RNA. As with the stable knockdown of NBS1, we observed significantly elevated levels of cGAS-positive micronuclei in tetracycline-mediated NBS1 knockdown cells in response to 6-thio-dG relative to shSCR cells treated with 6-thio-dG (*P* ≤ 0.0.01–0.001; Figure 2C). Furthermore, we noticed increased phosphorylation of IRF3, as assessed by western blotting (Figure 2D), significantly elevated levels of IL-6 secretion, as measured by ELISA (*P* ≤ 0.00001; Figure 2E), and significantly higher expression levels of genes associated with immune signaling, as quantified by qRT-PCR approach (*P* ≤ 0.01; Figure 2F), in shNBS1 (stable) cells relative to cells expressing control shRNA. Additionally, we detected a significantly higher number of β-galactosidase–positive cells in stable and tetracycline-inducible NBS1 knockdown samples than in the control shRNA-expressing samples in response to 6-thio-dG (*P* ≤ 0.01–0.02; Figure 2G).

We validated these results by using patient-derived NBS1-deficient NBS cells and NBS cells complemented with wild-type NBS1 (NBS + WT NBS1) (38). Initially, we noticed a significantly higher frequency of micronuclei in NBS cells than in NBS + WT NBS1 cells in response to 6-thio-dG (*P* ≤ 0.001; Figure 2H). Subsequently, we found that a significantly higher percentage of micronuclei had cGAS in cells lacking NBS1 than in NBS + WT NBS1 cells (*P* ≤ 0.01; Figure 2I). Furthermore, IRF3 activation, as assessed by western blotting (Figure 2J), was markedly higher in NBS-deficient cells than in NBS + WT NBS1 cells in response to 6-thio-dG. Additionally, NBS1-deficient cells exhibited significantly higher levels of senescence than NBS + WT NBS1 cells in response to 6-thio-dG (*P* ≤ 0.01; Figure 2K). Collectively, these findings reveal that defects in NBS1 result in an increase in cGAS-positive micronuclei, IRF3 phosphorylation, expression of immune genes, and senescence.

Subsequently, we evaluated whether NBS1 localization is restricted to micronuclei whose nuclear envelope is intact or ruptured (cytosolic chromatin fragments). Co-immunostaining with anti-NBS1 and anti-Lamin A/C antibodies revealed that ∼20% of NBS1-positive micronuclei are also positive for Lamin A/C, and the rest are devoid of any Lamin A/C staining (Supplementary Figure S2A). We corroborated these results by co-staining with anti-NBS1 and anti-RB1 antibodies. As with Lamin A/C–NBS1 co-localization, only 10–15% of micronuclei are positive for RB1 and NBS1, and the remaining NBS1-positive micronuclei are devoid of RB1 signals (Supplementary Figure S2B). To rule out the possibility that the higher number of cGAS-positive micronuclei resulted from a defective nuclear envelope coating, we counted the nuclear envelope–positive and -negative cGAS-positive micronuclei in shSCR RNA and shNBS1 RNA cells. We found that the number of nuclear envelope–coated cGAS-positive micronuclei in shNBS1 cells was comparable to that of shSCR cells (*P* ≤ 0.1; Supplementary Figure S2C). Therefore, the higher percentage of cGAS-positive micronuclei in NBS1 knockdown and NBS cells is not due to defects in the nuclear envelope coating, but to NBS1 deficiency.

### ATM and CtIP binding domains of NBS1 are critical for regulating cGAS recruitment to micronuclear DNA

**Figure 3.**
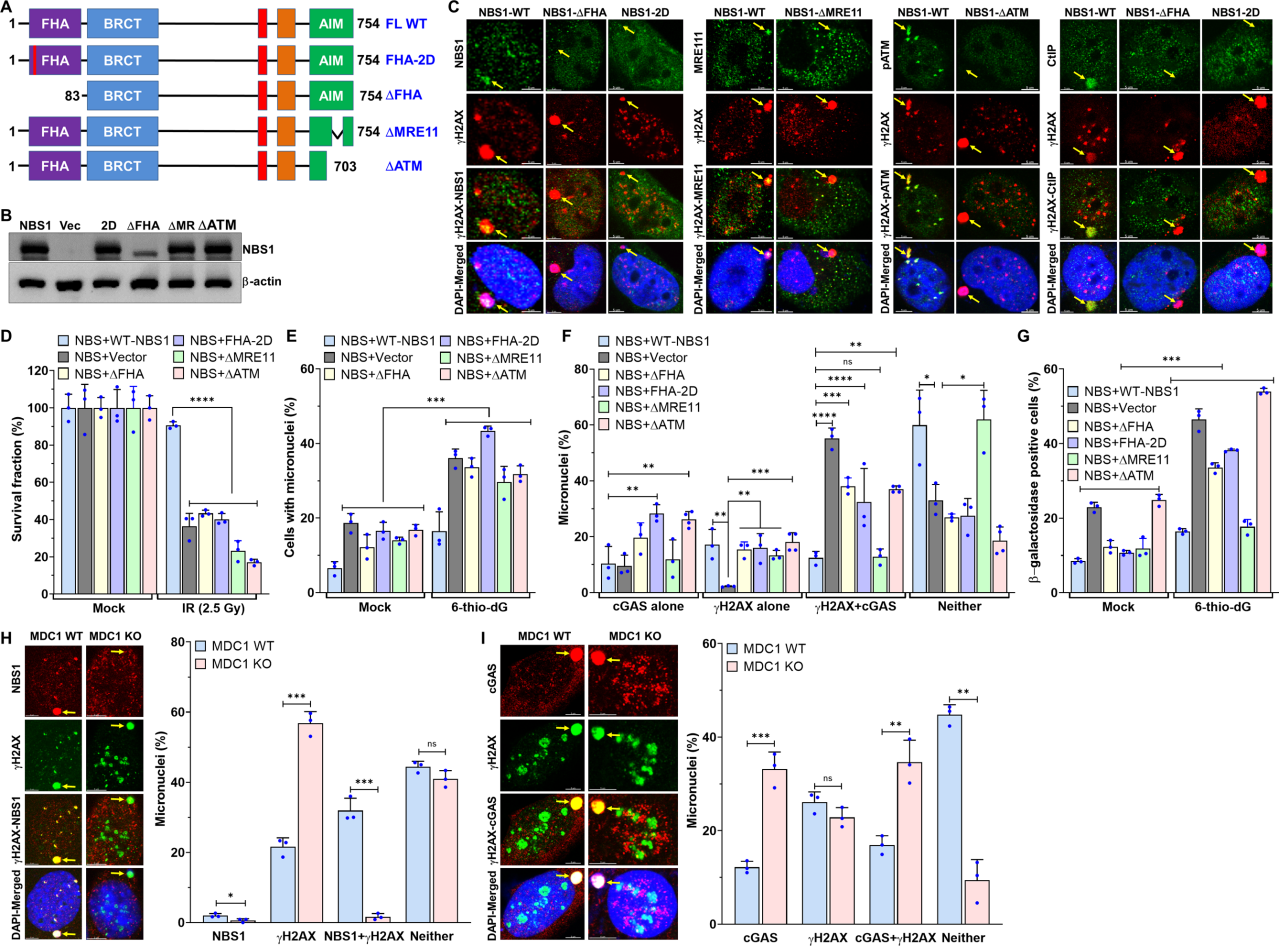
ATM- and CtIP-binding domains of NBS1 are critical for regulating cGAS binding to micronuclear DNA. (A, B) Schematics of different NBS1 mutant constructs used in this study (**A**). Representative western blot shows stable expression of different NBS1 mutants in patient-derived NBS cells (**B**). FHA–fork head-associated domain; BRCT–BRCA1/2 C-terminus domain; AIM–ATM-MRE11 interaction domain; FL-WT–full length wild-type; Vec–vector alone; ΔFHA–delta FHA; ΔMR–delta MRE11 and 2D-FHA–2D. (**C**) ΔFHA- and FHA-2D-mutant NBS1 not only failed to bind to micronuclei but also failed to recruit CtIP to micronuclei; ΔMRE11-NBS1 does not cause the translocation of MRE11 from the cytoplasm to the nuclear compartment, and ΔATM-NBS1 does not phosphorylate ATM in micronuclei. Representative confocal images show recruitment of NBS1 and its binding partners to micronuclei in NBS cells stably expressing WT- and ΔFHA-, FHA-2D-, ΔMRE11- and ΔATM- mutant-NBS1. Exponentially growing cells were exposed to 2.5 Gy ionizing radiation (IR) and subjected to immunostaining with indicated antibodies at 24 h after IR. (**D**) NBS cells stably expressing ΔFHA-, FHA-2D-, ΔMRE11- and ΔATM-mutant NBS1, but not WT-NBS1, are sensitive to ionizing radiation. Exponentially growing cells were exposed to 2.5 Gy ionizing radiation and allowed to form colonies for 10 days. Colonies were stained with Crystal violet solution, and the surviving fraction was calculated in three independent experiments. (E–G) Defects in the FHA and ATM-binding domains of NBS1 cause increased numbers of cGAS-positive micronuclei and senescent cells. Bar graph shows percentages of micronuclei in NBS cells stably expressing FHA-2D-, ΔFHA-, ΔMRE11- and ΔATM-NBS1 mutants at 72 h after mock- and 5 μM 6-thio-dG treatment (**E**). Bar graph shows the percentage of micronuclei harboring either cGAS alone, γH2AX alone, cGAS and γH2AX or neither in NBS cells stably expressing FHA-2D-, ΔFHA-, ΔMRE11- and ΔATM-NBS1 mutants at 72 h after 5 μM 6-thio-dG treatment (**F**). NBS cells stably expressing FHA-2D-, ΔFHA- and ΔATM-NBS1 mutants show higher levels of β-galactosidase staining than NBS + WT-NBS1 cells 7 days after 5 μM 6-thio-dG treatment (**G**). Bar graph presents the mean and STDEV from three independent experiments. Statistical analysis was performed using two-way ANOVA (E and F) and Student's *t*-test (G). (H, I) NBS1 is rarely recruited to micronuclei in the absence of MDC1: Representative images show the presence of NBS1 and γH2AX in the same micronucleus of MDC1 wild type (WT) but not in MDC1 knockout (KO) cells (**H**, left). Bar graph shows the frequency of micronuclei harboring either NBS1 alone, γH2AX alone, NBS1 and γH2AX or neither (**H**, right). Representative images show the presence of cGAS and γH2AX in the same micronucleus of MDC1 WT and in MDC1 KO cells (**I**, left) and the bar graph shows the frequency of micronuclei harboring either γH2AX alone, cGAS alone, γH2AX and cGAS or neither (I) in MDC1 WT and KO mouse embryonic fibroblasts 24 h after 2.5 Gy ionizing radiation. Bar graph presents the mean and STDEV from three independent experimental groups. * *P* ≤ 0.05; ** *P* ≤ 0.01; *** *P* ≤ 0.001; **** *P* ≤ 0.0001.

To identify the NBS1 domain that modulates cGAS binding to micronuclei, we used NBS cells stably expressing a panel of NBS1 mutants, including full-length (FL)-NBS1 harboring two mis-sense point mutations (Gly 27 Asp and Arg 28 Asp; FHA-2D) in the FHA domain, and FHA (ΔFHA; Δ1–82 amino acids)-, ATM recruitment domain (ΔATM; Δ703–754 amino acids)- and MRE11 binding domain (ΔMRE11; Δ682–693 amino acids)-deleted NBS1 (Figure 3A, B). Although the functions of different NBS1 domains in the primary nuclear compartment have been well-studied (28–32,51), we further characterized these mutants’ ability to be recruited to micronuclei (FHA-2D and ΔFHA) and to recruit binding partners, specifically ATM (ΔATM) and CtIP (ΔFHA) to micronuclei and MRE11 (ΔMRE11) to the primary nuclear compartment (Figure 3C). As with findings in the primary nuclear compartment, ΔFHA- and FHA-2D-NBS1 mutants failed to be recruited to the micronuclei (Figure 3C) (30). MRE11 was retained in the cytoplasm of NBS cells stably expressing ΔMRE11-NBS1 (Figure 3C), as described previously (30). Similar to previous reports in the primary nuclear compartment (52), we could not detect any phosphorylated ATM signal in the micronuclei of NBS and NBS cells stably expressing ΔATM-NBS1 (Figure 3C). As with previous *in vitro* and *in vivo* findings (34), CtIP was not recruited to the micronuclei of ΔFHA-NBS1 cells (Figure 3C). Subsequently, we evaluated the sensitivity of these NBS cells expressing different NBS1 mutants to ionizing radiation by using a colony formation assay. Similarly to previous findings (30), NBS cells complemented with different NBS1 mutants were significantly sensitive to ionizing radiation as compared with NBS cells complemented with WT-NBS1 (*P* ≤ 0.0001; Figure 3D). Thus, these results agree with previous findings on the functionality of different NBS1 mutants in the primary nuclear compartment, so we used these mutants for our subsequent studies.

Initially, we observed that the number of micronuclei was significantly higher in the NBS1 mutant cells treated with 6-thio-dG than in the mock-treated cells (*P* ≤ 0.01; Figure 3E). Interestingly, we found that the levels of cGAS-positive micronuclei were significantly higher in NBS cells expressing ΔFHA-, FHA-2D- and ΔATM-NBS1, but not in ΔMRE11-NBS1, than in NBS cells expressing WT-NBS1 (*P* ≤ 0.05–0.0001; Figure 3F). Additionally, we noticed that the percentage of β-galactosidase–positive NBS cells expressing ΔFHA-, FHA-2D- and ΔATM-NBS1, but not in ΔMRE11-NBS1, was significantly higher than in NBS cells expressing WT-NBS1 in response to 6-thio-dG (*P* ≤ 0.001; Figure 3G). Thus, the FHA domain-, ATM- and CtIP-binding domains, but not the MRE11-binding domain, of NBS1 may be involved in modulating cGAS binding to those micronuclear DNA. Because the FHA domain of NBS1 interacts with MDC1 (53,54), we assessed whether NBS1 binding to micronuclear DNA depends on MDC1. We detected significantly reduced number of NBS1-positive micronuclei in MDC1 knockout cells as compared with MDC1-WT cells (*P* ≤ 0.03–0.0001; Figure 3H). However, the number of cGAS-positive micronuclei was significantly higher in MDC1 knockout cells than in MDC1 WT cells (*P* ≤ 0.004–0.0007; Figure 3I), suggesting a role for MDC1-mediated recruitment of NBS1 in regulating cGAS binding to the micronuclei.

### NBS1-dependent recruitment of ATM is important for regulating cGAS binding to micronuclear DNA

**Figure 4.**
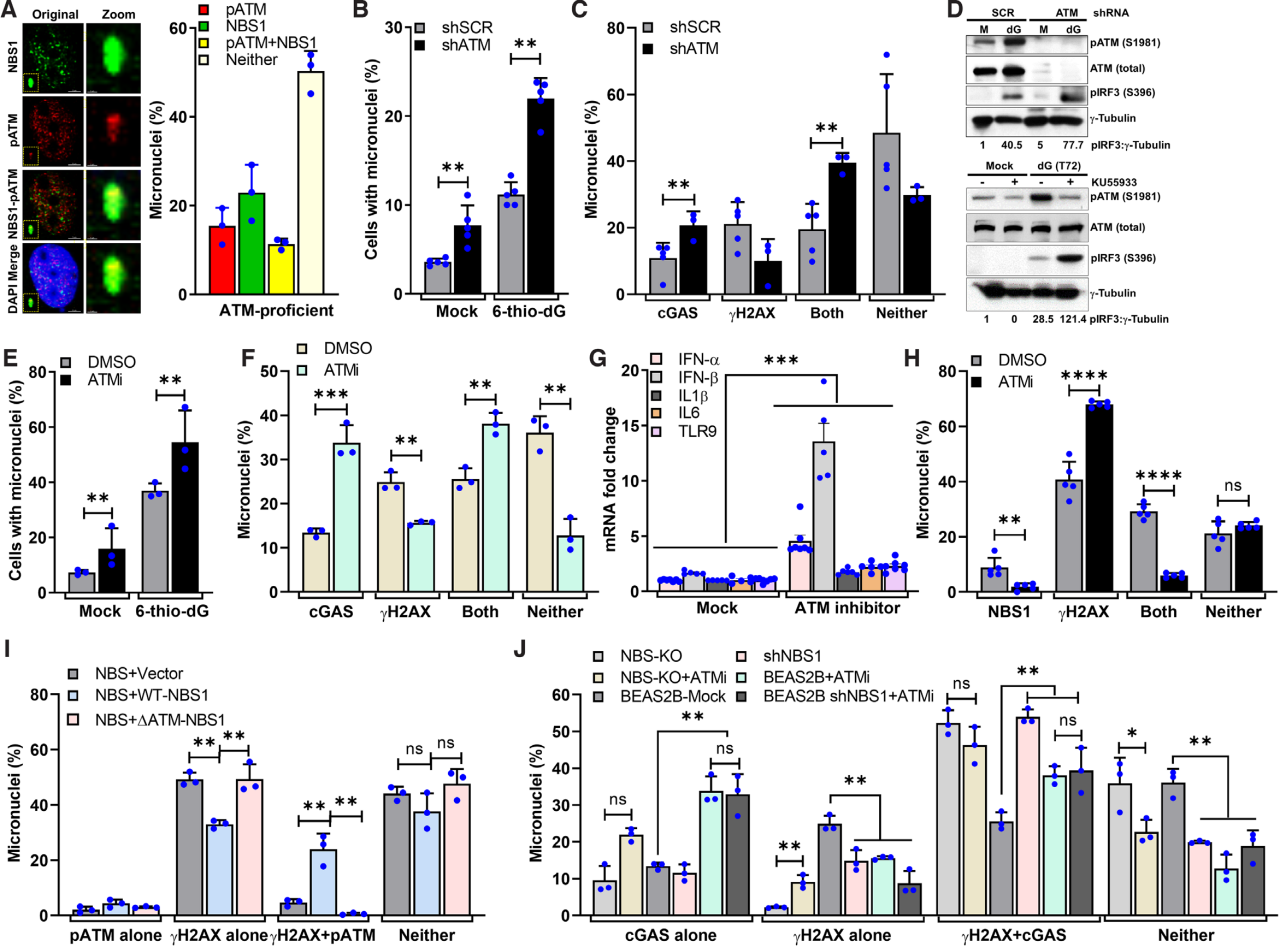
NBS1-mediated ATM recruitment to micronuclear DNA attenuates cGAS binding. (**A**) NBS1 co-localizes with phosphorylated ATM in micronuclei. Representative images show co-localization of NBS1 and phosphorylated ATM (S1981; left). Bar graph shows the frequency of micronuclei harboring either NBS1 alone, pATM alone, NBS1 and pATM or neither in BEAS2B cells treated with 6-thio-dG (72 h). Bar graph presents the mean and STDEV from three independent experiments. (B–D) ATM knockdown increases cGAS-positive micronuclei. Bar graph shows the frequency of micronuclei formation in mock- and 3 μM 6-thio-dG–treated shSCR and shATM cells (**B**). Bar graph shows the frequency of micronuclei harboring either cGAS alone, γH2AX alone, cGAS and γH2AX or neither in SCR shRNA- and ATM shRNA-expressing cells 72 h after 3 μM 6-thio-dG treatment (**C**). Bar graph presents the mean and STDEV from three independent experimental groups. Statistical analysis was performed using Student's *t*-test. ** *P* ≤ 0.01; *** *P* ≤ 0.001; **** *P* ≤ 0.0001. Representative western blot shows higher levels of IRF3 phosphorylation in shRNA-mediated ATM knockdown HeLa cells than in SCR shRNA-expressing HeLa cells (**D**, top) and in ATM inhibitor (KU55933)-treated cells than in DMSO-treated BEAS2B cells (**D**, bottom) exposed to 3 μM 6-thio-dG (dG) for 72 h. (E–G) Inhibition of ATM kinase activity enhances cGAS recruitment to micronuclear DNA. Bar graphs show the frequency of micronuclei formation (**E**), elevated levels of cGAS-positive micronuclei (**F**) and expression of IFN-α, IFN-β, IL1-α, IL6 and TLR9 (**G**) in cells treated with ATM kinase inhibitor (KU55933) as compared with DMSO-treated cells. Bar graph presents the mean and STDEV from three independent experiments. Statistical analysis was performed using Student's *t*-test. (**H**) Inhibiting ATM kinase activity reduces NBS1 recruitment to micronuclei. Bar graph shows the percentage of micronuclei containing either γH2AX alone, NBS1 alone, γH2AX and NBS1 or neither in DMSO- and ATM inhibitor (KU55933)-treated BEAS2B cells exposed to 3 μM 6-thio-dG for 72 h. Bar graph presents the mean and STDEV from three independent experiments. Statistical analysis was performed using Student's *t*-test. (**I**) Number of phosphorylated ATM–positive micronuclei is attenuated in NBS + Vector and NBS+ΔATM NBS1 cells. Bar graph shows the frequency of micronuclei harboring either pATM alone, γH2AX alone, pATM and γH2AX or neither in NBS + Vector, NBS + WT-NBS1 and NBS+ΔATM-NBS1 cells at 24 h after exposure to 2.5 Gy IR. Bar graph presents the mean and STDEV from three independent experiments. Statistical analysis was performed using Student's *t*-test. (**J**) Loss of both NBS1 and ATM functions is epistatic with respect to cGAS accumulation at micronuclei. Bar graph shows the percentage of micronuclei containing either cGAS alone, γH2AX alone, cGAS+γH2AX or neither in DMSO- and ATM inhibitor (KU55933)-treated BEAS2B + shNBS1 and NBS cells at 24 h after exposure to 2.5 Gy IR. Bar graph presents the mean and STDEV from three independent experiments. Statistical analysis was performed using Student's *t*-test. * *P* ≤ 0.05; ** *P* ≤ 0.01; *** *P* ≤ 0.001; **** *P* ≤ 0.0001.

Evidence shows that NBS1 recruits ATM to DSB sites via its C-terminal region (703–754 amino acids) in the primary nuclei (52) and that ATM deficiency enhances innate immune signaling (55–60). Additionally, we have found that NBS cells expressing NBS1 lacking ATM-recruitment domain (Δ703–754 or ΔATM) exhibited higher numbers of cGAS-positive micronuclei than WT-NBS1 cells (Figure 3F). To evaluate ATM’s role in modulating cGAS binding to micronuclear DNA, we initially examined the co-localization of NBS1 and ATM in micronuclei and found that a major fraction of NBS1 co-localized with phosphorylated ATM (S1981) in the micronuclei (Figure 4A). Subsequently, we found that shRNA-mediated ATM knockdown significantly increased micronuclei formation in mock-treated cells relative to mock-treated shSCR cells (*P* ≤ 0.001); micronuclei formation was further increased in ATM knockdown cells in response to 6-thio-dG as compared with mock-treated shATM cells (*P* ≤ 0.0001; Figure 4B). Furthermore, ATM knockdown cells exhibited significantly higher numbers of cGAS-positive micronuclei (*P* ≤ 0.01; Figure 4C) and enhanced IRF3 phosphorylation (Figure 4D, top).

To determine whether ATM’s kinase activity is important for modulating cGAS binding to micronuclear DNA, we inhibited this activity by using a small molecule inhibitor, KU55933. As with the ATM knockdown cells, we not only observed significantly increased micronuclei formation but also significantly greater numbers of cGAS-positive micronuclei in KU55933-treated cells than in DMSO-treated cells (*P* ≤ 0.01–0.001; Figure 4E, F). Additionally, we detected significantly higher expression levels of immune pathway genes (*P* ≤ 0.001; Figure 4G) and activation of IRF3 (Figure 4D, bottom) in the group treated with ATM inhibitor than in the group treated with DMSO. Subsequently, we examined the number of NBS1-positive micronuclei after inhibiting ATM kinase activity. In agreement with previous findings in the primary nuclei (29), we noticed that the number of NBS1-positive micronuclei in response to 6-thio-dG was significantly reduced upon inhibiting ATM kinase activity as compared with DMSO-treated samples (*P* ≤ 0.002–0.0001; Figure 4H). Furthermore, we observed that the numbers of pATM-positive micronuclei were significantly lower in NBS cells and NBS cells stably expressing ΔATM-NBS1 than in NBS cells stably expressing WT-NBS1 (*P* ≤ 0.001; Figure 4I).

To strengthen our assertion that NBS1 recruits ATM to micronuclei to prevent cGAS binding to micronuclear DNA, we determined whether the losses of NBS1 and ATM are epistatic with respect to cGAS binding to micronuclear DNA. For this purpose, we inhibited ATM kinase activity with KU55933, in BEAS2B-shNBS1 and NBS cells and examined cGAS recruitment to micronuclei. We found that the number of cGAS-positive micronuclei in ATM inhibitor–treated BEAS2B-shRNA and NBS cells was comparable to that in BEAS2B-shNBS1, NBS cells and ATM inhibitor alone groups (Figure 4J). These results suggest that the loss of both NBS1 and ATM functions is epistatic with respect to cGAS binding to micronuclear DNA. Collectively, NBS1-mediated recruitment of ATM plays a role in regulating cGAS binding to the micronuclear DNA.

### cGAS is not recruited to end-resected micronuclear DNA

**Figure 5.**
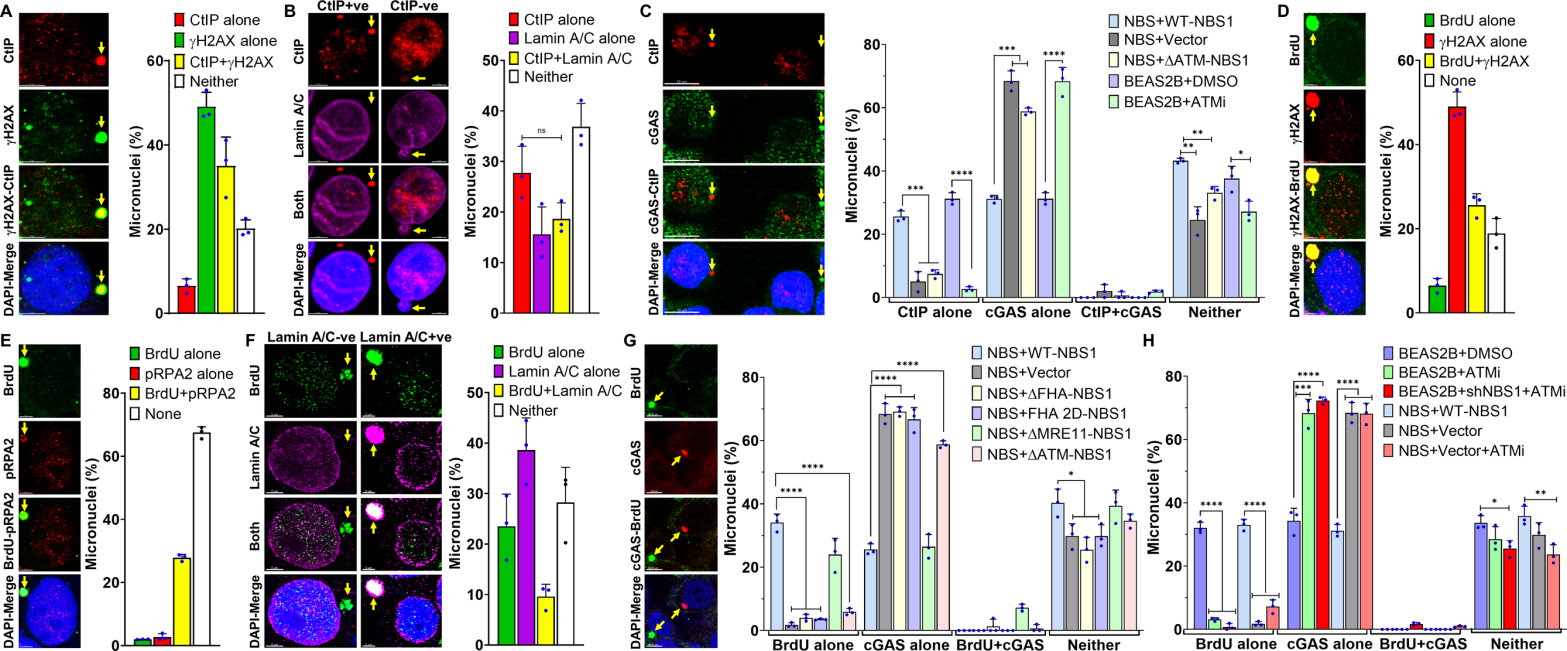
CtIP-positive and end-resected micronuclear DNA are defective in cGAS binding. (A–C) CtIP is recruited to micronuclei but does not co-localize with cGAS. Representative images show co-localization of CtIP with γH2AX (**A**, left), Lamin A/C (**B**, left) and cGAS (**C**, left) in the micronuclei. Bar graphs show the frequency of micronuclei harboring either CtIP alone, γH2AX alone, CtIP and γH2AX or neither (**A**, graph) and CtIP alone, Lamin A/C alone, CtIP and Lamin A/C or neither (**B**, graph) in BEAS2B cells at 24 h after exposure to 2.5 Gy IR. Bar graph shows the frequency of micronuclei harboring either CtIP alone, cGAS alone, CtIP and cGAS or neither in NBS + Vector, NBS + WT NBS1, NBS+ΔATM NBS1, BEAS2B + DMSO and BEAS2B + ATM inhibitor (10 μM KU55933) cells at 24 h after exposure to 2.5 Gy IR (**C**, graph). Bar graphs present the mean and STDEV from three independent experiments. (D–G) Most micronuclear DNA with end-resected ssDNA are negative for cGAS. Representative images show co-localization of end-resected ssDNA marker (BrdU) with γH2AX (**D**, left), pRPA2 (**E**, left), Lamin A/C (**F**, left) and cGAS (**G**, left) in the micronuclear DNA. Bar graphs show the frequency of micronuclear DNA harboring either BrdU alone, γH2AX alone, BrdU and γH2AX or neither (D, graph); BrdU alone, pRPA2 alone, BrdU and pRPA2 or neither (E, graph); BrdU alone, Lamin A/C alone, BrdU and Lamin A/C or neither (F, graph) in BEAS2B cells at 24 h after exposure to 2.5 Gy IR. Bar graph shows the frequency of micronuclei harboring either BrdU alone, cGAS alone, BrdU and cGAS or neither in NBS and NBS cells stably expressing ΔFHA-, FHA-2D-, ΔMRE11- and ΔATM-NBS1 mutants (G, graph). Bar graphs present the mean and STDEV from three independent experiments. Statistical analysis was performed using Student's *t*-test. (**H)** Inhibition of ATM kinase activity limits micronuclear DNA end resection, and this is epistatic with NBS1. Bar graphs show the frequency of micronuclei harboring either BrdU alone, cGAS alone, BrdU and cGAS or neither in BEAS2B-shSCR + DMSO, BEAS2B-shSCR + ATM inhibitor, BEAS2B-shNBS1 + ATM inhibitor, NBS-WT NBS1 + DMSO, NBS-Vector + DMSO and NBS-Vector + ATM inhibitor cells at 24 h after exposure to 2.5 Gy IR. Bar graphs present the mean and STDEV from three independent experiments. Statistical analysis was performed using Student's *t*-test. * *P* ≤ 0.05; ** *P* ≤ 0.01; *** *P* ≤ 0.001; **** *P* ≤ 0.0001.

Next, we set out to determine how NBS1 modulates cGAS binding to micronuclear DNA. Because ΔFHA-NBS1 and ΔATM-NBS1 cells exhibited higher numbers of cGAS-positive micronuclei than WT-NBS1 cells (Figure 3F), and because these domains are responsible for recruiting CtIP and RNF20, respectively, to DSBs in the primary nuclear compartment, we evaluated the involvement of CtIP and RNF20 in cGAS recruitment to micronuclei. First, we verified the recruitment and co-localization of CtIP with cGAS in the micronuclei. As predicted, CtIP was recruited to the micronuclear DNA and clearly co-localized with the γH2AX foci (Figure 5A). Next, we detected CtIP in both Lamin A/C–positive and negative micronuclei (Figure 5B), which indicates that CtIP is recruited to both nuclear envelope–positive and -negative micronuclei, albeit to a varying degree. Subsequently, we evaluated the relationship between CtIP and cGAS recruitment to micronuclear DNA. Interestingly, we found that a major fraction of CtIP-positive micronuclei was negative for cGAS, similar to NBS1 (Figure 5C). As expected, the number of CtIP-positive micronuclei was significantly lower in NBS1 knockdown cells than in NBS1-proficient cells (*P* ≤ 0.001; Figure 5C), which confirms a role for NBS1 in recruiting CtIP to micronuclei (34). Additionally, we verified CtIP recruitment in NBS+ΔATM NBS1 and ATM inhibitor–treated cells and found that CtIP recruitment to micronuclear DNA is significantly lower in NBS, NBS+ΔATM NBS1, BEAS2B + ATM inhibitor–treated cells than in NBS + WT NBS1 and DMSO control groups (*P* ≤ 0.001–0.0001; Figure 5C). Next, we examined the recruitment and co-localization of RNF20 with cGAS in the micronuclei. We rarely detected an RNF20 signal in the micronuclei, whether by indirect immunostaining or by live cell imaging (data not shown), which rules out a possible role for RNF20 in modulating cGAS binding to micronuclei. Thus, CtIP is recruited to micronuclear DNA in an NBS1-dependent manner, and it does not co-localize with cGAS in the same micronuclear DNA.

CtIP resects DSBs, and that generates ssDNA overhangs in the primary nuclear compartment (36). Therefore, first, we examined micronuclear DNA end resection by using a non-denaturing BrdU immunostaining approach, as described previously (36). We detected a prominent BrdU signal, representing end-resected DNA, in ∼35% of micronuclei. We also found that a major fraction of BrdU signal-positive micronuclei are also positive for γH2AX (Figure 5D). Second, we examined the co-localization of RPA2, a component of the tripartite Replication Protein A complex, often used as a surrogate readout for ssDNA (61–65), with BrdU signal. We detected co-localization of phosphorylated RPA2 (Serine 33; pRPA2) and BrdU signal in micronuclei (Figure 5E), which confirms that DNA end resection occurs in the micronuclear DNA. Third, we found BrdU signal in both nuclear envelope–positive (∼10%) and negative (∼23%) micronuclei (Figure 5F), which indicates that DNA end resection takes place in both micronuclei and cytosolic chromatin fragments, albeit to a varying degree. Subsequently, we evaluated the relationship between DNA end resection and cGAS recruitment in micronuclei. Surprisingly, as with lack of co-localization of cGAS with NBS1 and CtIP, a major fraction of micronuclei positive for BrdU signal were negative for cGAS signal (Figure 5G, images and graphs). To establish a link between NBS1 binding partners, cGAS recruitment and micronuclear DNA end resection, we evaluated the frequency of DNA end resection in the micronuclei of NBS cells expressing various NBS1 mutants. We found that the number of BrdU signal–positive micronuclei was significantly reduced in NBS and NBS cells stably expressing ΔFHA-, FHA 2D-, and ΔATM-NBS1, but not in WT-NBS1 and ΔMRE11-NBS1 cells, as compared with NBS + WT-NBS1 cells (*P* ≤ 0.0001; Figure 5G).

To determine whether inhibiting ATM also restricts end resection in micronuclei, and whether this is epistatic with NBS1 depletion, we inhibited ATM kinase activity with KU55933, in BEAS2B-shSCR, BEAS2B-shNBS1, NBS + vector and NBS + WT NBS1 cells and examined BrdU signals in micronuclei. We found that the number of BrdU signal–positive micronuclei in BEAS2B-shSCR cells treated with ATM inhibitor was significantly lower than in the mock-treated BEAS2B-shSCR group (*P* ≤ 0.0001; Figure 5H). Additionally, we noticed that the number of BrdU signal–positive micronuclei in BEAS2B-shNBS1, NBS and ATM inhibitor alone treated cells was comparable to that in the BEAS2B-shNBS1 and NBS + vector cells treated with ATM inhibitor (Figure 5H). These results suggest that both NBS1 and ATM functions are epistatic with respect to end resection of micronuclear DNA. Overall, DNA end resection takes place in the micronuclear DNA, NBS1 and its binding partners influence micronuclear DNA end resection, and cGAS does not bind to a major fraction of end-resected micronuclear DNA.

### CtIP-catalyzed DNA end resection suppresses cGAS binding to micronuclear DNA

**Figure 6.**
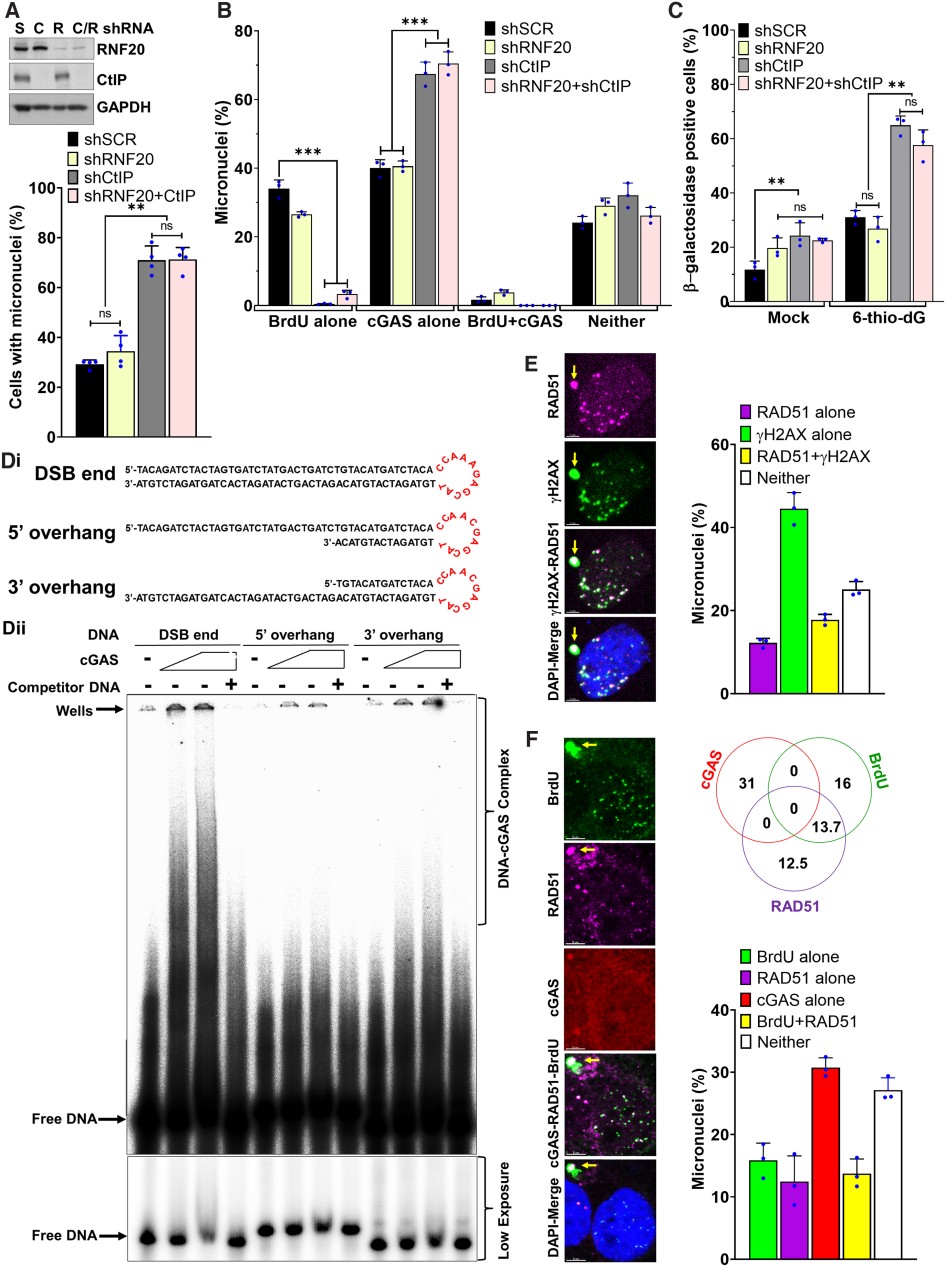
CtIP-mediated end resection attenuates cGAS binding to micronuclear DNA. (A–C) Downregulation of CtIP enhances cGAS-positive micronuclei. Representative western blots show expression of CtIP and RNF20 in BEAS2B cells stably expressing shSCR (S), shCtIP (C), shRNF20 (R) and shCtIP + shRNF20 (C/R) (**A**, top). Bar graphs show percentages of cells with micronuclei (A); percentages of micronuclei harboring either cGAS alone, BrdU alone, cGAS and BrdU or neither (**B**) in BEAS2B cells stably expressing scrambled (SCR), shRNF20, shCtIP and shRNF20 and shCtIP at 24 h after exposure to 2.5 Gy IR. Bar graph shows percentages of β-galactosidase–positive cells expressing scrambled (SCR), shRNF20, shCtIP and shRNF20 and shCtIP RNAs at 10 days after exposure to 2.5 Gy IR (**C**). Bar graph presents the mean and STDEV of three independent experiments. Statistical analysis was performed using two-way ANOVA. (**D**) cGAS binds with double-stranded but not end-resected DNA substrates. Schematic shows the three different DNA structures used for the *in vitro* cGAS binding assay (**i**). Representative phosphor-image shows cGAS binding with double-stranded but not with end-resected DNA substrates (**ii**). 5–10 μM cGAS was incubated with 50 fmol ^32^P–labeled DNA substrates in the presence or absence of 100 bp cold double-stranded DNA (competitor). DNA protein complex was resolved onto 5% native polyacrylamide gel electrophoresis, and the signal was detected by phosphor-imaging. (**E**) RAD51 is recruited to micronuclear DNA, and it co-localizes with γH2AX. Representative images show co-localization of RAD51 and γH2AX in the same micronuclei (left) and the bar graph shows the percentages of either RAD51 alone, γH2AX alone, RAD51 and γH2AX or neither in HT1080 cells at 24 h after exposure to 2.5 Gy IR. Data in the bar graph represent mean and STDEV from three independent experiments. (**F**) RAD51 and cGAS do not co-localize in the same micronuclei. Representative images show localization of cGAS, RAD51 and BrdU signal in the micronuclei of BEAS2B cells at 24 h after exposure to 2.5 Gy IR. Venn diagram and the bar graph show the percentages of micronuclei with indicated markers. Data in the Venn diagram and the bar graph represent mean and STDEV from three independent experiments. * *P* ≤ 0.05; ** *P* ≤ 0.01; *** *P* ≤ 0.001.

To verify the role of CtIP in mediating DNA end-resection in micronuclei, we depleted either CtIP alone or in combination with RNF20 (Figure 6A, inset), and examined both DNA end resection and cGAS recruitment to micronuclei. First, we detected significantly higher numbers of micronuclei in both shCtIP and shCtIP/shRNF20 groups than the shSCR and shRNF20 groups at 24 h after exposure to 2.5 Gy IR (*P* ≤ 0.01; Figure 6A). Second, we carried out BrdU labeling following by immunofluorescence with anti-BrdU antibodies under non-denaturing conditions and assessed DNA end resection in the micronuclei. As expected, similar to NBS1 knockdown cells, the number of BrdU signal–positive micronuclei was significantly lower in CtIP knockdown cells than in CtIP-proficient cells (*P* ≤ 0.001; Figure 6B). Third, we noticed that ∼70% of micronuclei in CtIP knockdown cells were positive for cGAS (Figure 6B). However, RNF20 depletion did not significantly alter the number of cGAS-positive micronuclei, as compared with shSCR cells (Figure 6B). Importantly, CtIP knockdown cells exhibited a significantly increased number of senescent cells as compared with CtIP-proficient cells after 2.5 Gy IR treatment (*P* ≤ 0.01; Figure 6C). Collectively, these findings show that CtIP–mediated end-resected DNA may not be a preferred binding substrate for cGAS.

DNA end resection in the primary nuclear compartment is initiated by the MRN complex, which functions in conjunction with CtIP (36,66). We evaluated the contribution of MRE11 and its exonuclease activity to modulating cGAS recruitment to micronuclei by using MRE11-depleted cells (shMRE11; Supplementary Figure S3A) and by treating MRE11-proficient cells with Mirin, a small molecule exonuclease inhibitor of MRE11. Surprisingly, we found that neither depleting MRE11 nor inhibiting its exonuclease activity increased the number of cGAS-positive micronuclei in response to 6-thio-dG (Supplementary Figure S3B-D and supplementary results). Furthermore, 6-thio-dG treatment did not increase the expression levels of immune pathway genes in MRE11-defective cells; rather, the expression of many genes was significantly reduced upon 6-thio-dG treatment in the shMRE11 and mirin-treated cells, as compared with MRE11-proficient cells (*P* ≤ 0.05–0.0001; Supplementary Figure S3E-G and supplementary results), as previously reported (67). Subsequently, we evaluated the contribution of MRE11 and its exonuclease activity to modulating DNA end resection in micronuclei by using BrdU labeling followed by immunostaining with anti-BrdU antibodies under non-denaturing conditions. We observed that neither depleting MRE11 nor inhibiting its exonuclease activity decreased the number of BrdU-positive micronuclei (Supplementary Figure S3H), which suggests that DNA end resection in micronuclei does not require MRE11. Additional experiments are needed to understand why MRE11 is not involved in DNA end resection in the micronuclei, despite its contributions to DNA end resection in the primary nuclear compartment. Nevertheless, we infer from these results that neither MRE11 nor its exonuclease activity plays a role in regulating cGAS recruitment to micronuclei.

Yet, it is not clear whether cGAS can bind to the end-resected ssDNA. We carried out in vitro experiments using purified cGAS and DNA substrates that mimic end-resected DNA (Supplementary Figure S4A-C). Similar to a previous study (46), cGAS bound strongly to a 45–base-pair (bp) double-strand (dsDNA) interferon stimulatory DNA substrate (Figure 6Di and Supplementary Figure S4D, E). However, cGAS binding to the same DNA substrate mimicking either 5′- or 3′-resected DNA was substantially reduced (Figure 6Dii). It is worth noting that the DNA substrate mimicking end-resected DNA that we used here has a 30-nucleotide overhang. This is smaller than the products of NBS1-CtIP-mediated end resection, which are generally resected 100 nucleotides and bound by RPA in cells. Therefore, additional experiments using longer resected DNA substrates (∼100 nucleotides) in the presence or absence of purified recombination factors (RPA2, NBS1, CtIP and RAD51) are needed to verify the contribution of recombination factors to cGAS binding to end-resected DNA in vitro.

Finally, we verified whether DNA end resection in micronuclei leads to RAD51, a central homologous recombination factor, recruitment. We found that RAD51 is not only co-localized with γH2AX (Figure 6E), but it also co-localized with the BrdU signal in the same micronuclei (Figure 6F). Additionally, as expected, CtIP knockdown inhibited RAD51 binding to micronuclei (data not shown). Surprisingly, like NBS1 and CtIP, we rarely detected co-localization of RAD51 and cGAS in the same micronuclei (Figure 6F). Taken together, these results provide strong evidence that the coordinated activities of NBS1 and CtIP convert micronuclear dsDNA ends to ssDNA ends, and this conversion prevents cGAS from binding to micronuclear DNA.

### cGAS is activated in a major fraction of micronuclei in NBS1- and CtIP-depleted cells

**Figure 7.**
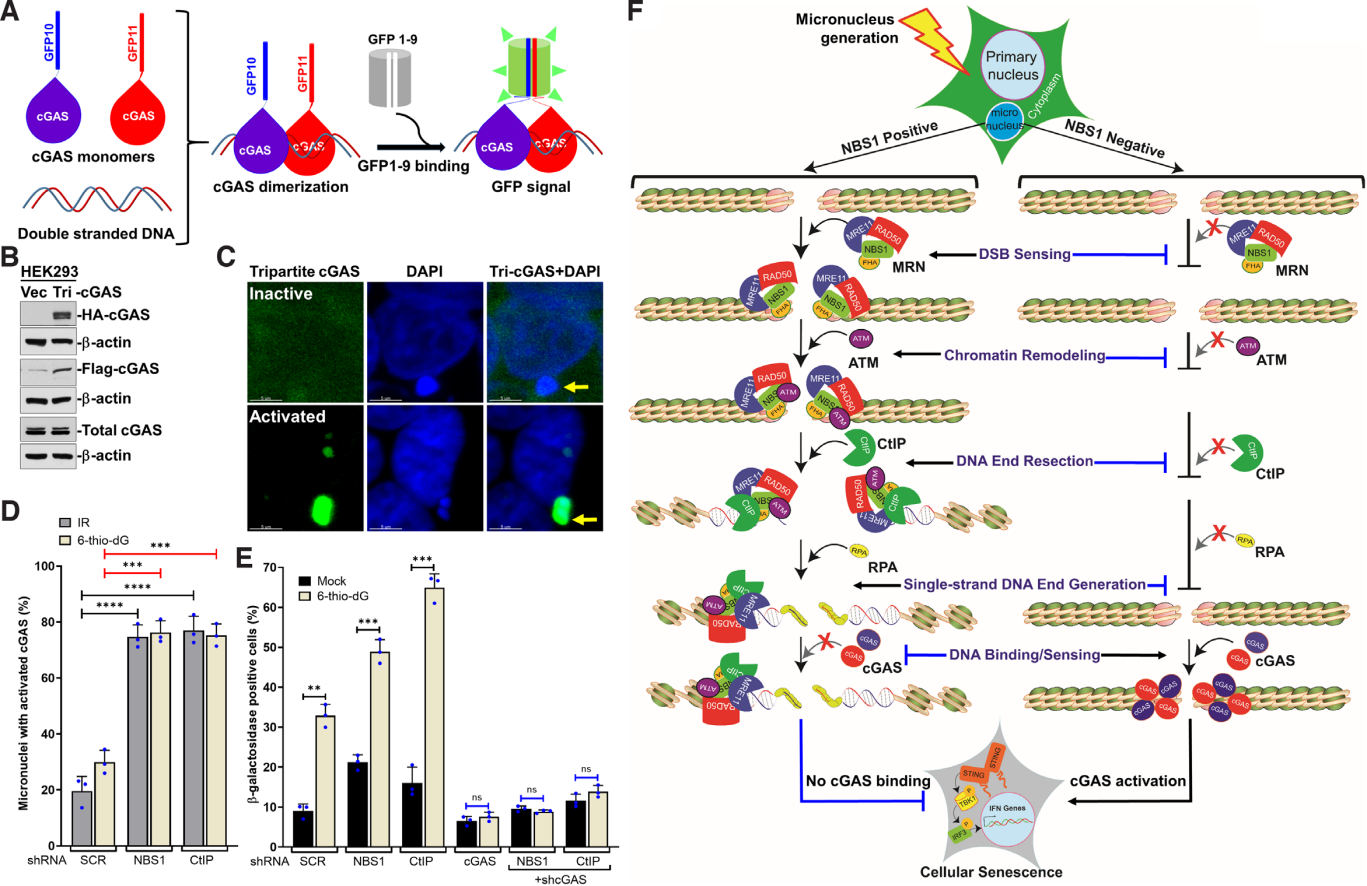
cGAS is activated in response to a major fraction of micronuclear DNA in NBS1- and CtIP-depleted cells. (**A**) Schematics show tripartite cGAS system consisting of G10-Flag-cGAS, cGAS-HA-G11 and GFP1-9 for detecting activated cGAS in cells. The principle is that when two different molecules of cGAS, i.e., G10-Flag-cGAS and cGAS-HA-G11, dimerize on double-stranded DNA, that binds to GFP1-9, which results in a functional GFP. (**B**) Western blots show detection of G10-Flag-cGAS and cGAS-HA-G11 expression in HEK 293 cells. Cells were infected with lentiviral particles carrying G10-Flag-cGAS, cGAS-HA-G11 and GFP1-9, then were selected with Puromycin, G418 and hygromycin. Total cell lysate was probed with anti-HA, anti-Flag-HRP, cGAS and β-actin antibodies. (C, D) cGAS is activated in a major fraction of micronuclear DNA in NBS1 and CtIP knockdown cells. Representative images show GFP fluorescence signal in the micronuclei triggered by the dimerized (i.e. activated) cGAS (**C**). Bar graph shows the percentages of activated cGAS-positive micronuclei in shSCR, shNBS1 and shCtIP cells at 24 h after 2.5 Gy ionizing radiation (IR) and at 72 h after 1 μM 6-thio-dG treatment (**D**). HEK293 cells stably expressing tripartite cGAS were transfected with shSCR, shNBS1 and shCtIP RNAs and treated with either 2.5 Gy IR or 1 μM 6-thio-dG, and the GFP signal in the micronuclei was analyzed at indicated times after the treatment. Bar graph presents the mean and STDEV from 300–400 cells in three independent experimental groups. Statistical analysis was performed using Student's *t*-test. (**E**) Co-depleting cGAS and NBS1, and cGAS and CtIP abrogates cellular senescence. Bar graph shows percentages of β-gal–positive cells expressing SCR, NBS1 alone, CtIP alone, cGAS alone, NBS1+cGAS and CtIP+cGAS shRNAs at 10 days after 3 μM 6-thio-dG (6-dG) treatment. Bar graph presents the mean and STDEV of three independent experiments. Statistical analysis was performed using Student's t-test. * *P* ≤ 0.05; ** *P* ≤ 0.01; *** *P* ≤ 0.001; **** *P* ≤ 0.0001. (**F**) Model depicting the mechanism of NBS1-mediated regulation of cGAS binding to micronuclei and the subsequent activation of immune signaling, which culminates in cellular senescence.

Although we detected elevated levels of cGAS-positive micronuclei in NBS1 and CtIP knockdown cells, it is unclear whether these cGAS represent its activated form. We examined the efficiency of cGAS activation upon its association with micronuclear DNA by using a newly developed cGAS tripartite system that detects activated cGAS in different cellular compartments (Figure 7A-C) (14). We noticed cGAS activation in a limited number of cytosolic chromatin fragments in samples treated with 6-thio-dG and ionizing radiation (Figure 7D). Depleting NBS1 significantly increased this number relative to shSCR samples (*P* ≤ 0.001–0.0001; Figure 7D). Similarly, we noticed cGAS activation in a significantly larger fraction of micronuclei in CtIP knockdown cells than in the control group (*P* ≤ 0.001–0.0001; Figure 7D). These results suggest that cGAS is not only recruited to a higher percentage of micronuclei in the absence of NBS1 and CtIP but is also activated in these micronuclei.

cGAS is essential for cellular senescence, but it is unclear whether the elevated levels of cellular senescence that we observed in NBS1 and CtIP knockdown cells responding to DNA damage are also mediated by cGAS. To investigate this idea, we co-knocked down NBS1 and cGAS, as well as CtIP and cGAS, in the same cells by shRNAs, caused genotoxic stress with 6-thio-dG, and counted β-gal–positive cells 10 days later. As shown in Figure 7E, the number of β-gal–positive cells in response to 6-thio-dG was significantly lower in co-knocked down cells than in cells depleted of NBS1 or CtIP alone (*P* ≤ 0.0001). Additionally, we noticed that the percentage of β-gal–positive cells in shNBS1-shcGAS and shCtIP-shcGAS cells was similar to that in cells depleted of cGAS alone (Figure 7E). Thus, cGAS is responsible for the increased cellular senescence we observed in NBS1 and CtIP knockdown cells.

## DISCUSSION

Here, we provide cell and molecular biology and biochemical evidence that cGAS is recruited to only a sub-set of micronuclei with ruptured envelope and that NBS1 inhibits cGAS binding to micronuclear DNA by promoting CtIP-mediated end resection. Evidence suggests that cGAS has a higher affinity for nucleosomes than for naked DNA (10) and for double-strand DNA (dsDNA) than for single-strand DNA (ssDNA; *K*_d_ ∼1.5 μM) (46,68). End resection mediated by NBS1-CtIP and their binding partners not only converts DSB ends into 3′ ssDNA overhangs, but also remodels (or relaxes) nucleosomes surrounding the resected DNA (69). We detected not only the recruitment of NBS1, ATM, CtIP and RPA2 to micronuclear DNA, but also accumulation of ssDNA in the micronuclear DNA. These observations clearly demonstrated not only the occurrence of micronuclear DNA end resection, but also that these micronuclear DNA lack cGAS, further confirming the inability of cGAS to interact with end-resected micronuclear DNA. Additionally, due to the inability of CtIP recruitment, the efficiency of micronuclear DNA end resection in ΔFHA-, FHA-2D- and ΔATM- NBS1 cells, but not in WT-NBS1 and ΔMRE11-NBS1 cells, is significantly reduced, resulting in the elevated levels of cGAS positive micronuclear DNA. All of these observations establish that the end-resected micronuclear DNA may not be a preferred cGAS binding substrate.

NBS1, together with MRE11 and RAD50 (MRN complex), was long thought to mainly sense DSBs and to transmit DNA damage signaling to regulate genomic integrity and cellular homeostasis. Our current findings suggest a novel role for this well-studied DDR factor in limiting cGAS recruitment to micronuclear DNA. NBS1 lacks any known enzymatic activities, but MDC1-dependent recruitment of NBS1 to damaged chromatin and NBS1’s subsequent recruitment of its binding partners ATM and CtIP to micronuclear DNA choreograph the conversion of dsDNA ends into ssDNA ends, and that conversion prevents cGAS from binding micronuclear DNA. We provide extensive evidence that: (i) Both transient and stable knockdown of NBS1 enhance the percentage of cGAS-positive micronuclear DNA. Because NBS1 recruits CtIP and ATM to DSB sites, NBS1 deficiency causes defects in CtIP-initiated DNA end resection (34), which results in the recruitment of cGAS to more micronuclear DNA. (ii) Expression of NBS1 defective in the C-terminal ATM binding domain (delta 705–754 aa) and inhibition of ATM kinase activity increased the number of cGAS-positive micronuclear DNA. ATM kinase activity is important for NBS1 and CtIP phosphorylation, as well as for CtIP activation (34,70). Defective ATM functioning attenuates DNA resection and enhances cGAS binding to micronuclear DNA. (iii) Expression of NBS1 defective in the CtIP-binding region (delta FHA) promoted cGAS recruitment to a major fraction of micronuclear DNA because of the reduced levels of DSB resection of the micronuclear DNA. (iv) Inhibition of ATM kinase activity with a small molecule ATM inhibitor (KU55933) in NBS and BEAS2B-shNBS1 cells did not alter the number of cGAS positive micronuclei as compared with either NBS1 depletion or ATM inhibition, suggesting that loss of both NBS1 and ATM are epistatic with respect to cGAS recruitment to micronuclear DNA. Overall, our findings demonstrate that the coordinated activities of NBS1 and its binding partners, specifically ATM and CtIP, alter the architecture of micronuclei, which prevents cGAS from binding micronuclear DNA.

CtIP is essential for DSB resection to generate 3′ single-stranded overhangs (36). CtIP is recruited to DSB sites by NBS1 through its N-terminal FHA-BRCT1/2 domains (34). Furthermore, NBS1-dependent recruitment of ATM phosphorylates CtIP and is also required for its nuclease activity in vitro (34,71). Since nucleosome organization forms a barrier for resection, NBS1-CtIP–dependent recruitment of nucleases/helicases (EXO1, DNA2, BLM and WRN) and chromatin remodeling factors (SMARCAD1 and INO80) remodel the chromatin organization and help to complete the DNA end resection process (72–76). Moreover, CtIP-dependent DSB resection is not limited to the S/G2 phases of the cell cycle: DSB resection has also been reported in the G0/G1 and M phases (61–65,77). Our observations on the recruitment of CtIP to micronuclear DNA, our detection of BrdU signals by a non-denaturing BrdU immunofluorescence approach and the presence of phosphorylated RPA2 in micronuclear DNA suggest that micronuclear DNA undergoes DSB resection. Additionally, depleting either NBS1 or CtIP not only reduced the resection of micronuclear DNA, as indicated by the reduction in BrdU signal–positive micronuclear DNA, but also enhanced cGAS binding to a major fraction of micronuclear DNA. In support of our findings, recent evidence shows that Bloom syndrome protein (BLM) deficiency increases cGAS-positive micronuclei and that TREX1-mediated resection of micronuclear DNA constrains cGAS activation in micronuclei (26,27). Thus, DNA end resection plays a key function in suppressing cGAS’s interaction with micronuclear DNA. It would be important in the future to understand how other DNA end resection nucleases (Exonuclease 1 and DNA2) and the chromatin remodelers associated with this process contribute to regulating cGAS binding to micronuclear DNA.

We have combined the data from this study and presented them as a model in Figure 7F. Following the generation of micronuclei in response to genotoxic stress, NBS1 is recruited to micronuclear DNA, probably via MDC1. Upon binding to micronuclear DNA, NBS1 serves as a docking platform for multiple proteins, including ATM and CtIP. Subsequently, CtIP resects dsDNA ends and converts them into ssDNA ends, which results in their being coated by the ssDNA binding protein RPA2. Because cGAS has a strong affinity for dsDNA but not for ssDNA, these resected micronuclear DNA may not be a strong binding substrate for cGAS; this prevents cGAS from binding to the micronuclear DNA with end-resected DSBs. In the absence of any micronuclear DSB end resection—whether due to defective NBS1, ATM or CtIP—cGAS is not only recruited to micronuclear DNA, but is also activated upon binding to the micronuclear dsDNA. This initiates innate immune signaling, which culminates in a senescence phenotype. In summary, cGAS does not bind to all micronuclei with ruptured nuclear-envelope; and by promoting CtIP-mediated end resection, NBS1 inhibits cGAS binding to micronuclear DNA.

## DATA AVAILABILITY

The data that support the findings of this study are available from the corresponding author upon reasonable request.

## Supplementary Material

gkac079_Supplemental_File
