# Supplementary material for: NBS1-CtIP–mediated DNA end resection suppresses cGAS binding to micronuclei
Source: Nucleic Acids Res. 2022 Feb 22;50(5):2681–99. doi: 10.1093/nar/gkac079 (PMC8934670; doi:10.1093/nar/gkac079)
Supplement: gkac079_Supplemental_File [file gkac079_supplemental_file.pdf]

Nucleic Acids Research

Supplemental Information

NBS1-CtIP–Mediated DNA End Resection Suppresses cGAS Binding to Micronuclei

Salim Abdisalaam[#,1], Shibani Mukherjee[#,*,1], Souparno Bhattacharya[#,1], Sharda Kumari[1], Debapriya Sinha[1], Janice Ortega[1], Guo-Min Li[1] , Hesham A. Sadek[2], Sunil Krishnan[3] and Aroumougame Asaithamby[*,1]

[#]These authors contributed equally to this work.

[1]Department of Radiation Oncology, [2]Department of Internal Medicine, University of Texas Southwestern Medical Center, Dallas, Texas 75390

[3]Department of Radiation Oncology, Mayo Clinic, Jacksonville, Florida 32082

*Corresponding authors

Division of Molecular Radiation Biology<

Department of Radiation Oncology

University of Texas Southwestern Medical Center

Dallas, Texas 75390

Phone: 214-648-5175

Email: Asaithamby.Aroumougame@UTSouthwestern.edu

Shibani.Mukherjee@UTSouthwestern.edu

Fax: 214-648-5995

Running title: NBS1 regulates cGAS binding to micronuclei

Keywords: NBS1, cGAS, CtIP, ATM, DNA damage response, micronuclei, end resection

Supplementary Figure Legends

Figure S1: MDC1, but not MRE11, co-localizes with cGAS in micronuclei.

A. Representative images show co-localization of MDC1 and cGAS in micronuclei. Bar graphs show the frequency of micronuclei harboring either MDC1 alone, cGAS alone, MDC1 and cGAS or neither in cells expressing DN-TRF2 and treated with doxycycline for 72 hours (**right**).

B. Representative images show no (left) or weak co-localization of MRE11 with cGAS in micronuclei. Bar graphs show the frequency of micronuclei harboring either MRE11 alone, cGAS alone, MRE11 and cGAS or neither in cells expressing DN-TRF2 and treated with doxycycline for 72 hours (**right**). Bar graph presents the mean and STDEV from three independent experiments.

Figure S2: **NBS1 is recruited to both nuclear envelope-positive and -negative micronuclei and the increased proportion of cGAS-positive micronuclei in NBS1 knockdown and NBS cells is not due to micronuclei's defective nuclear envelope coating.**

A. NBS1 recruitment to micronuclei is independent of nuclear envelope coating. Bar graph shows the frequency of micronuclei containing either nuclear envelope marker (Lamin A/C) alone, NBS1 alone, Lamin A/C and NBS1 or neither in BEAS2B cells treated with 3 μM 6-thio-dG. Data in the bar graph present the mean and STDEV from three independent experiments.

B. Only a minor fraction of NBS1 co-localizes with RB1 onto micronuclei. Bar graph shows the percentage of micronuclei harboring RB1, NBS1, both or neither in BEAS2B cells treated with 3 μM 6-thio-dG. Data in the bar graph present the mean and STDEV from three independent experiments.

C. Increased proportion of cGAS-positive micronuclei in NBS1 defective cells is not due to micronuclei's defective nuclear envelope coating. Bar graphs show the percentage of micronuclei harboring either Lamin A/C coating (NE) alone, cGAS alone, Lamin A/C and cGAS or neither in BEAS2B cells stably expressing shSCR- and doxycycline-inducible shNBS1 RNAs at 72 hours after 3 μM 6-thio-dG treatment. Bar graphs present the mean and STDEV from three independent experiments. Statistical analysis was performed using Student's t-test.

Figure S3: **Neither MRE11 nor its exonuclease activity play a role in micronuclear DNA end resection and cGAS recruitment to micronuclear DNA.**

A-E. MRE11 deficiency and blocking its exonuclease activity increase the number of micronuclei, but not immune signaling. Western blot shows MRE11 expression in HT1080 cells stably expressing shMRE11 RNA and ALTD (MRE11-mutant) cells (**A**). Bar graph shows

frequency of micronuclei formation in MRE11-depleted (HT1080+shMRE11) and MRE11 exonuclease-inhibited (HT1080+mirin) cells at 72 hours after 3 μM 6-thio-dG withdrawal (**B**). Data in the bar graph present the mean and STDEV from 3-4 independent experiments. Bar graphs show the percentages of micronuclei harboring either cGAS, γH2AX, cGAS and γH2AX or neither in MRE11-depleted HT1080 cells (**C**) and Mirin-treated HT1080 cells (**D**) 72 hours after 3 μM 6-thio-dG treatment. Data in the bar graph represent STDEV from three to four independent experiments.

E-G. Expression of immune pathway genes is reduced in MRE11-defective cells. Bar graph shows expression of immune pathway genes in MRE11-proficient HT1080 cells (**E**), MRE11-depleted HT1080 cells (**F**), and mirin-treated HT1080 cells (**G**) at 72 hours after 3 μM 6-thio-dG treatment. Error bars represent the STDEV from three-five independent experiments.

H. Micronuclear DNA end resection is not affected by MRE11. Bar graph shows the percentage of micronuclei with either cGAS alone, BrdU signal alone, cGAS and BrdU signal or neither in HT1080-shSCR, HT1080+shMRE11 and HT1080+Mirin treated cells at 24 hours after exposure to 2.5 Gy IR. Data in the bar graph represent STDEV from three independent experiments. * P (range) $\leq$0.05; **** P$\leq$0.0001.

Figure S4: Purification of human cGAS and its binding to double-strand break ended DNA substrate but not to DNA substrate harboring resected DNA ends.

A. Coomassie brilliant blue stained SDS-PAGE shows cGAS elution from Histidine column.

B. Coomassie brilliant blue stained SDS-PAGE shows presence of cGAS after elution from Heparin column with 0.5-1M NaCl gradient.

C. Coomassie brilliant blue stained SDS-PAGE shows cGAS purity after elution from Superdex 200 column.

D. Schematic representation of three different DNA structures used for cGAS-DNA binding assay.

E. cGAS binds with double-stranded but not to end-resected DNA substrates. 5-10 μM cGAS was incubated with 25 fmol ^{32}P labeled DNA substrates in the presence of absence of 100 base pairs cold double stranded DNA (competitor). DNA-Protein complex was resolved onto 5% native Poly acrylamide gel electrophoresis and the signal was detected by phosphor imaging.

Supplementary Tables

Table S1: List of primers used for cloning small hairpin RNAs and cGAS binding assay reported in this study.

Table S2: List of primary antibodies and their respective dilutions used for both western blotting (WB) and immunofluorescence staining (IF) reported in this study.

Table S3: List of primers used for qRT-PCR reported in this study.

Supplementary Results

MRE11 does not influence cGAS recruitment to micronuclei: Once NBS1 senses DSBs via its FHA-BRCT1/2 domains, it recruits multiple proteins, including MRE11,[1] ATM,[2,3] and CtIP,[4-6] to these DSBs for chromatin remodeling, end-processing and downstream DDR signaling. Although ΔMRE11-NBS1 expression in NBS cells did not alter the number of cGAS-positive micronuclei, a previous report indicated a role for MRE11 in sensing cytosolic DNA fragments.[7] However, our results on the lack of co-localization between MRE11 and cGAS within the micronuclei and the cytosolic localization of MRE11 prompted us to investigate MRE11's involvement in the accumulation of cGAS in micronuclei. First, we depleted MRE11 in HT1080 cells by using MRE11-specific shRNA (**Fig. S3A**). We found that depleting MRE11 did not alter the number of micronuclei that formed in response to 6-thio-dG treatment as compared with control group (**Fig. S3B**). Furthermore, the accumulation of cGAS in the micronuclei in shMRE11 cells was comparable to that of shSCR cells (**Fig. S3C**). Additionally, similar to a previous report,[7] the expression of immune signaling genes was reduced in the absence of MRE11 (**Figs. S3E-G**).

Second, since MRE11 possesses both exonuclease and endonuclease activities, we used mirin to inhibit MRE11's exonuclease activity to further show that blocking this activity does not augment cGAS accumulation in the micronuclei. As with MRE11 depletion, pre-treating cells with mirin and then with 6-thio-dG did not alter cGAS recruitment to the micronuclei as compared with DMSO-treated cells (**Fig. S3D**). Additionally, mirin treatment significantly reduced the expression of genes involved in immune pathways in response to 6-thio-dG treatment as compared with 6-thio-dG treated DMSO cells (**Fig. S3G**). So, MRE11 depletion prevents the induction of an inflammatory response (**Figs.S3E-G**) even though cGAS recruitment to micronuclei is not altered (**Fig. S3D**). Thus, cGAS recruited to a limited number of micronuclei in MRE11 depleted cells but does not trigger robust immune response.

Supplementary References

1 Tauchi, H. *et al.* The forkhead-associated domain of NBS1 is essential for nuclear foci formation after irradiation but not essential for hRAD50[middle dot]hMRE11[middle dot]NBS1 complex DNA repair activity. *J Biol Chem* **276**, 12-15, doi:10.1074/jbc.C000578200 (2001).

2 Lim, D. S. *et al.* ATM phosphorylates p95/nbs1 in an S-phase checkpoint pathway. *Nature* **404**, 613-617, doi:10.1038/35007091 (2000).

3 Wu, X. *et al.* ATM phosphorylation of Nijmegen breakage syndrome protein is required in a DNA damage response. *Nature* **405**, 477-482, doi:10.1038/35013089 (2000).

4 Spycher, C. *et al.* Constitutive phosphorylation of MDC1 physically links the MRE11-RAD50-NBS1 complex to damaged chromatin. *The Journal of cell biology* **181**, 227-240, doi:10.1083/jcb.200709008 (2008).

5 Wang, H. *et al.* The interaction of CtIP and Nbs1 connects CDK and ATM to regulate HR-mediated double-strand break repair. *PLoS Genet* **9**, e1003277, doi:10.1371/journal.pgen.1003277 (2013).

6 Williams, R. S. *et al.* Nbs1 flexibly tethers Ctp1 and Mre11-Rad50 to coordinate DNA double-strand break processing and repair. *Cell* **139**, 87-99, doi:10.1016/j.cell.2009.07.033 (2009).

7 Kondo, T. *et al.* DNA damage sensor MRE11 recognizes cytosolic double-stranded DNA and induces type I interferon by regulating STING trafficking. *Proceedings of the National Academy of Sciences of the United States of America* **110**, 2969-2974, doi:10.1073/pnas.1222694110 (2013).

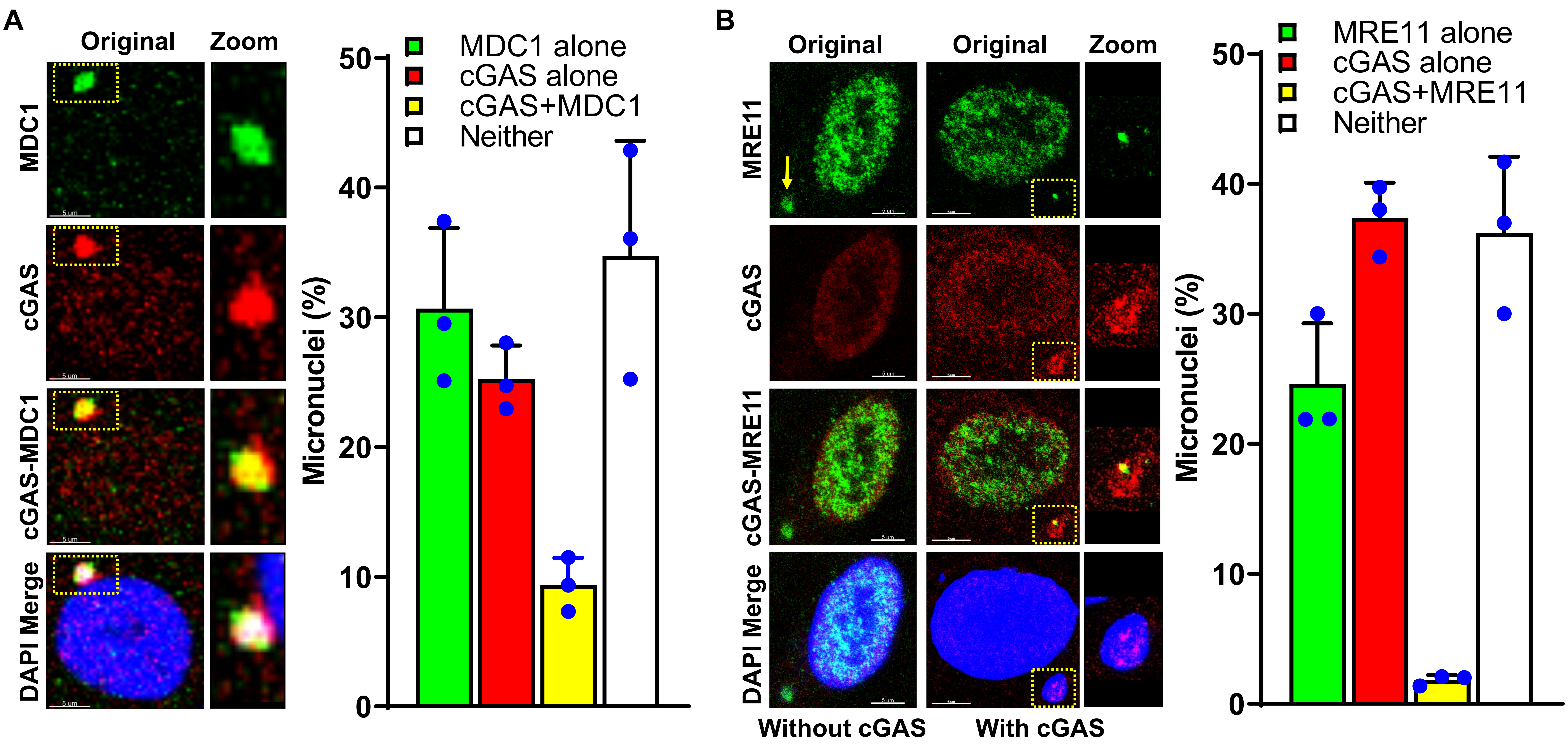
A
Original
Zoom
MDC1
cGAS
cGAS-MDC1
DAPI Merge
MDC1 alone
cGAS alone
cGAS+MDC1
Neither
Micronuclei (%)
0
10
20
30
40
50
B
Original
Original
Zoom
MRE11
cGAS
cGAS-MRE11
DAPI Merge
Without cGAS
With cGAS
MRE11 alone
cGAS alone
cGAS+MRE11
Neither
Micronuclei (%)
0
10
20
30
40
50

Figure S2

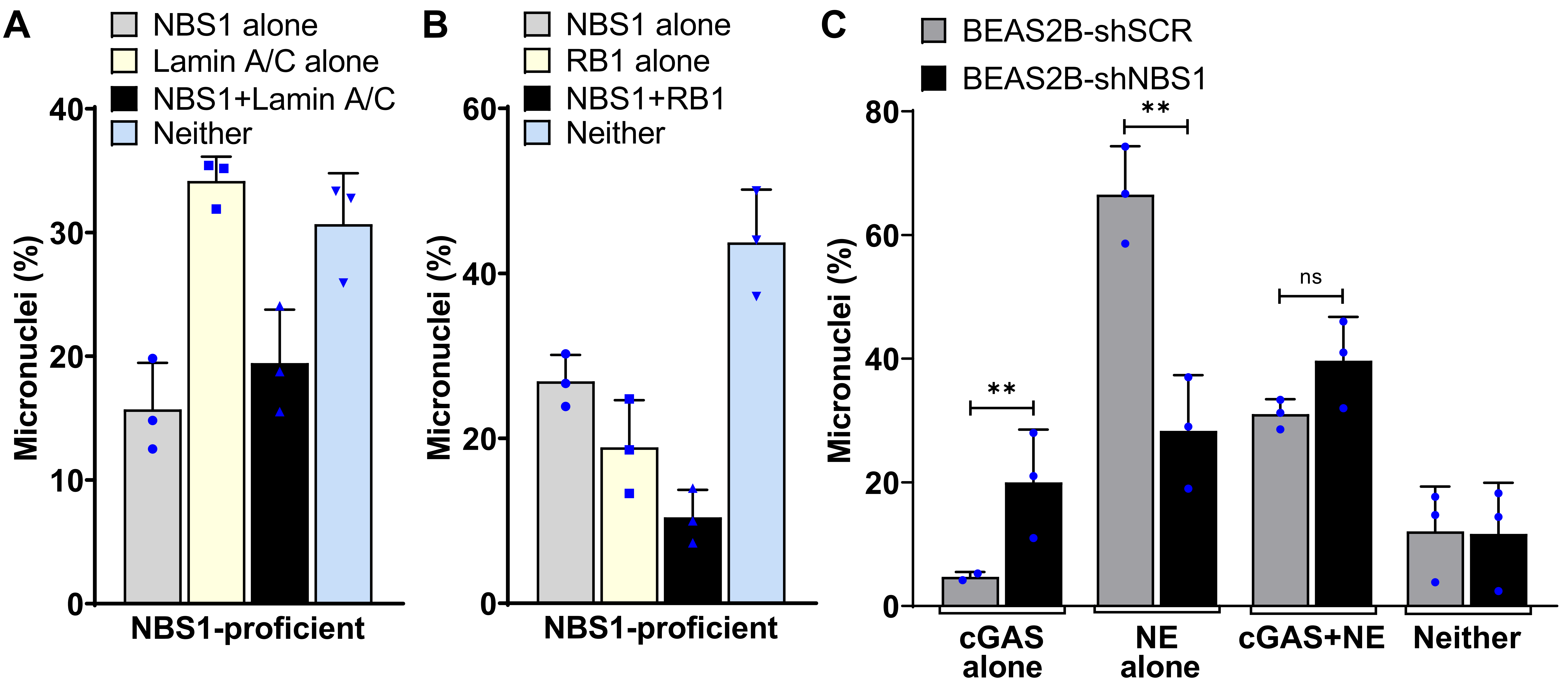
A
NBS1 alone
Lamin A/C alone
NBS1+Lamin A/C
Neither
Micronuclei (%)
0
10
20
30
40
NBS1-proficient
B
NBS1 alone
RB1 alone
NBS1+RB1
Neither
Micronuclei (%)
0
20
40
60
NBS1-proficient
C
BEAS2B-shSCR
BEAS2B-shNBS1
Micronuclei (%)
0
20
40
60
80
**
**
ns
cGAS alone
NE alone
cGAS+NE
Neither

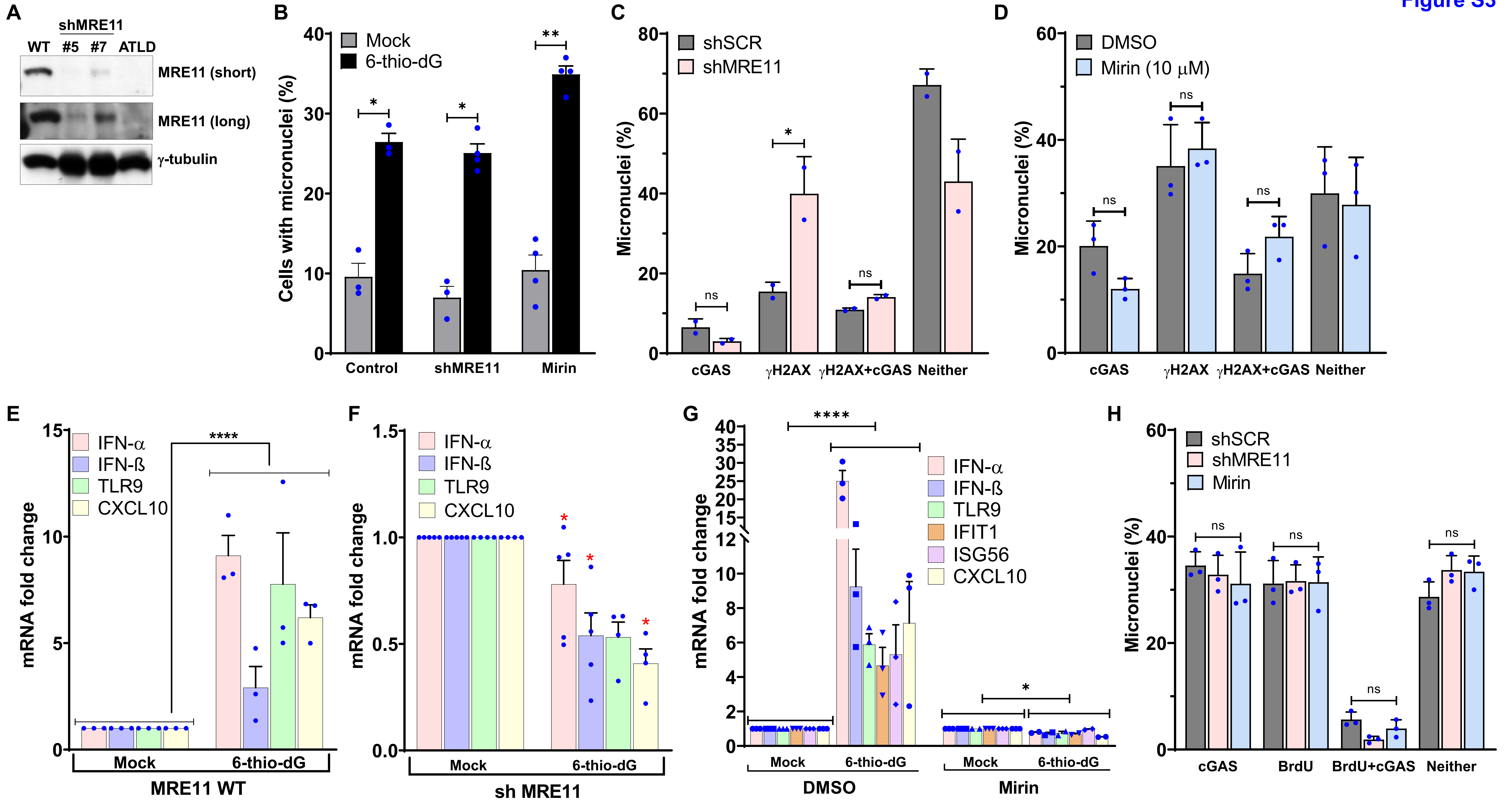
A
shMRE11
WT
#5
#7
ATLD
MRE11 (short)
MRE11 (long)
γ-tubulin
B
Cells with micronuclei (%)
Mock
6-thio-dG
Control
shMRE11
Mirin
C
Micronuclei (%)
shSCR
shMRE11
cGAS
γH2AX
γH2AX+cGAS
Neither
D
DMSO
Mirin (10 μM)
E
mRNA fold change
IFN-α
IFN-ß
TLR9
CXCL10
Mock
6-thio-dG
MRE11 WT
F
sh MRE11
G
IFIT1
ISG56
DMSO
Mirin
H
shSCR
shMRE11
Mirin
BrdU
BrdU+cGAS
Neither

Figure S4

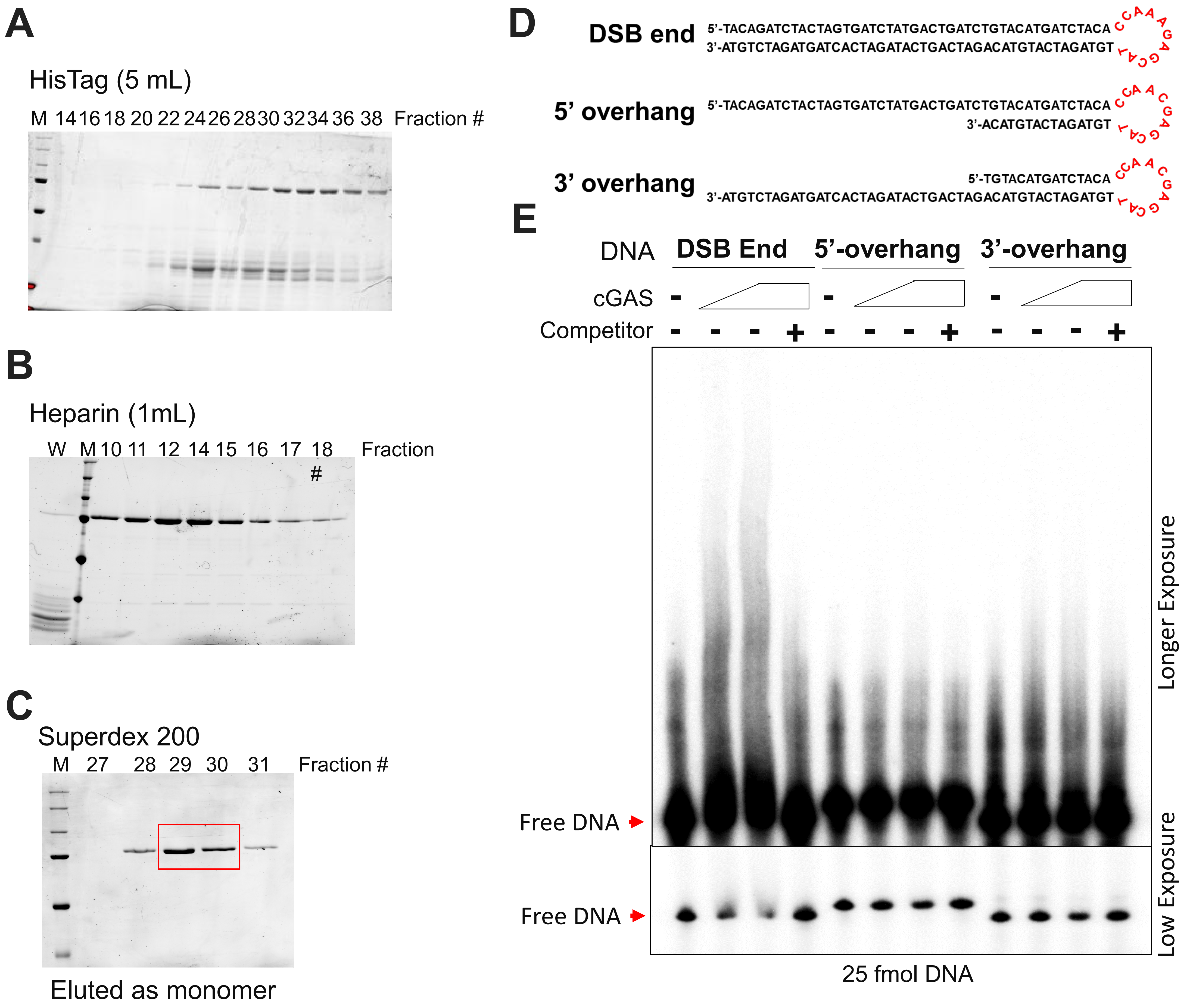

A
HisTag (5 mL)
M 14 16 18 20 22 24 26 28 30 32 34 36 38 Fraction #
B
Heparin (1mL)
W M 10 11 12 14 15 16 17 18 Fraction #
C
Superdex 200
M 27 28 29 30 31 Fraction #
Eluted as monomer
D
DSB end
5'-TACAGATCTACTAGTGATCTATGACTGATCTGTACATGATCTACA
3'-ATGTCTAGATGATCACTAGATACTGACTAGACATGTACTAGATGT
5' overhang
5'-TACAGATCTACTAGTGATCTATGACTGATCTGTACATGATCTACA
3'-ACATGTACTAGATGT
3' overhang
5'-TGTACATGATCTACA
3'-ATGTCTAGATGATCACTAGATACTGACTAGACATGTACTAGATGT
E
DNA
DSB End
5'-overhang
3'-overhang
cGAS
Competitor
Free DNA
Free DNA
Longer Exposure
Low Exposure
25 fmol DNA

Table S1: List of Primers used for cloning and EMSA

Serial #	Primer Name	Sequence (5'-3')
1	NBS1-shRNA1-F1	CCGG CCATCCCAGTACAGGATTAAACTCGAGTTTAATCCTGTACTGGGATGG TTTTT
2	NBS1shRNA1-R1	AATT AAAAA CCATCCCAGTACAGGATTAAACTCGAGTTTAATCCTGTACTGGGATGG
3	NBS1-shRNA2-F2	CCGG CCTCTTGATGAACCATCTATTCTCGAGAATAGATGGTTCATCAAGAGG TTTTT
4	NBS1shRNA2-R2	AATT AAAAA CCTCTTGATGAACCATCTATTCTCGAGAATAGATGGTTCATCAAGAGG
5	NBS1-shRNA3-F3	CCGG GCTTATTTAGAGTCCTAGTTTCTCGAGAAACTAGGACTCTAAATAAGC TTTTT
6	NBS1shRNA3-R3	AATT AAAAA GCTTATTTAGAGTCCTAGTTTCTCGAGAAACTAGGACTCTAAATAAGC
7	DSB End substrate	TACAGATCTACTAGTGATCTATGACTGATCTGTACATGATCTACA**ccaaagagcagTGTAGATCATGTACAGATCAGTCATAGATCACTAGTAGATCTGTA**
8	5'-Resected substrate	TACAGATCTACTAGTGATCTATGACTGATCTGTACATGATCTACA**ccaaagagcagTGTAGATCATGTACA**
9	3'-Resected substrate	TGTACATGATCTACA**ccaaagagcagTGTAGATCATGTACAGATCAGTCATAGATCACTAGTAGATCTGTA**

Table S2: List of antibodies used in this study

Sl. No.	Primary Antibodies	Catalog #	Vendor	Application (dilution)
1	Mouse monoclonal anti-γ-Tubulin (GTu88)	T6557	Sigma	WB (1:50000)
2	Rabbit monoclonal anti-phospho-IRF3 (Ser396;4D4G)	4947	Cell Signaling	WB (1:500)
3	Mouse monoclonal anti-cGAS	242363	Abcam	IF (1:100)
4	Rabbit monoclonal anti-cGAS (D1D3G)	15102	Cell Signaling	WB (1:200); IF (1:100)
5	Mouse monoclonal anti-MDC1 (MDC1-50)	50003	Abcam	IF (1:400)
6	Rabbit polyclonal anti-phospho-ATM (Ser1981; EP1890Y)	81292	Abcam	WB (1:10000); IF (1:500)
7	Mouse monoclonal anti-phospho Stat1 (pSTAT1-Tyr 701; A-2)	8394	Santacruz	WB (1:200)
8	Mouse monoclonal anti-Lamin A/C (636)	7292	Santacruz	IF (1:300)
9	Mouse monoclonal anti-NBS1	NB100-221	Novus Bio	IF (1:100), WB (1:500)
10	Rabbit polyclonal anti-NBS1	3001	Cell Signaling	IF (1:100)
11	Rabbit polyclonal anti-NBS1	NB100-143	Novus Bio	WB (1:500); IF (1:100)
12	Mouse monoclonal anti-MRE11 (12D7)	70212	Gentex	WB (1:1000); IF (1:1000)
13	Mouse monoclonal anti-ATM (2C1)	70103	Gentex	WB (1:5000)
14	Mouse monoclonal anti-phospho-Histone H2AX (Ser139; JBW301)	05-636	Millipore	IF (1:1000)
15	Mouse monoclonal anti-RNF20	89007928	Abnova	IF (1:100)
16	Rabbit Polyclonal CtIP	38016	Abcam	IF (1:100)
17	Rabbit polyconal phosphorylated RPA2	NA	homemade	IF (1:100)
18	Mouse monoclonal RPA2	NA	homemade	IF (1:100)
19	Rat monoclonal anti-BrdU	NB500-169	Novus Biologicals	IF (1:100)
20	Rabbit monoclonal anti-β-Actin (13E5)	4970	Cell Signaling	WB (1:1000)

Table S3

Serial #	Primer Name	Sequence
1	human IFNα Fwd (5'-3')	AACTCCCCTGATGAATGCGG
2	human IFNα Rev (5'-3')	TAGCAGGGGTGAGAGTCTTTG
3	human IFNβ Fwd (5'-3')	CAACTTGCTTGGATTCCTACAAAG
4	human IFNβ Rev (5'-3')	TATTCAAGCCTCCCATTCAATTG
5	human TLR9 Fwd (5'-3')	CGCCCTGCACCCGCTGTCTCT
6	human TLR9 Rev (5'-3')	CGGGGTGCTGCCATGGAGAAG
7	human IL1β Fwd (5'-3')	CCACCACTACAGCAAGGG
8	human IL1β Rev (5'-3')	GAACTGGGCAGACTCAAA
9	human CXCL10 Fwd (5'-3')	AGGAACCTCCAGTCTCAGCA
10	human CXCL10 Rev (5'-3')	CAAAATTGGCTTGCAGGAAT
11	human IFIT1 Fwd (5'-3')	CCTCCTTGGGTTCGTCTACA
12	human IFIT1 Rev (5'-3')	GGCTGATATCTGGGTGCCTA
13	human IFIT3 Fwd (5'-3')	GAAGGAACTGGGCCGCCTGCTAAG
14	human IFIT3 Rev (5'-3')	GCCCTGGCCCATTTCCTCACTACC
15	human ISG56 Fwd (5'-3')	TTGATGACGATGAAATGCCTGA
16	human ISG56 Rev (5'-3')	CAGGTCACCAGACTCCTCAC
17	human IL6 Fwd (5'-3')	CCTTCGGTCCAGTTGCCTTCT
18	human IL6 Rev (5'-3')	GCATTTGTGGTTGGGTCA
19	human β-Actin Fwd (5'-3')	TCGTCGACAACGGCTCCGGCATGT
20	human β-Actin Rev (5'-3')	CCAGCCAGGTCCAGACGCAGGAT
